# The association between diet and bladder cancer risk: a two-sample mendelian randomization

**DOI:** 10.1007/s00394-025-03743-5

**Published:** 2025-06-18

**Authors:** Ya-nan Dai, Evan Yi-Wen Yu, Maurice P. Zeegers, Anke Wesselius

**Affiliations:** 1 Department of Epidemiology, School of Nutrition and Translational Research in Metabolism, Maastricht University, P. Debyeplein 1 (Room A2.037), 6229ER Maastricht, the Netherlands; 2 Key Laboratory of Environmental Medicine and Engineering of Ministry of Education, School of Public Health, Southeast University, Nanjing, 210009 China; 3 Department of Epidemiology & Biostatistics, School of Public Health, Southeast University, Nanjing, 210009 China

**Keywords:** Bladder cancer, Mendelian randomization, Diet, Dietary patterns, GWAS

## Abstract

**Purpose:**

Bladder cancer (BC) is the ninth most common cancer globally, with notable gender and regional differences. Due to the inconclusive impact of diet on BC risk and the inherent biases from observational studies, this research investigates the causal role of dietary patterns (DPs) in BC risk using Two-Sample Mendelian Randomization (TSMR).

**Methods:**

Genetic variants were used as instrumental variables (IVs) to analyze 14 food/beverage items and four DPs. Summary-level exposure and outcome data were derived from UK Biobank GWAS (>500,000 participants). The inverse-variance weighted (IVW) method was the primary approach, supported by sensitivity tests including weighted median, MR-Egger regression, and MR-PRESSO.

**Results:**

No causal links were identified between BC risk and 14 specific foods or two DPs (Low-Caloric and Acquired Diet Patterns). However, greater adherence to PC1 (high in whole grains, fruits, and vegetables) was associated with a reduced BC risk (OR: 0.74, 95% CI 0.61–0.89, p < 0.05). In contrast, the Highly Palatable Dietary Pattern (HPDP), rich in processed and high-calorie foods, was linked to a more than twofold increase in BC risk (OR: 2.81, 95% CI 1.43–5.52, p < 0.05). These findings were consistent across sensitivity analyses, with no evidence of pleiotropy or heterogeneity.

**Conclusions:**

Adherence to PC1 may lower BC risk, while HPDP may increase it, emphasizing the relevance of overall dietary habits in BC prevention. Further research in diverse populations is recommended to develop more targeted prevention strategies and better translate findings into practice for improved BC-related public health outcomes.

**Supplementary Information:**

The online version contains supplementary material available at 10.1007/s00394-025-03743-5.

## Introduction

Bladder cancer (BC) is the ninth most common cancer worldwide, with approximately 614,000 new cases and 220,000 deaths occurring in 2022, accounting for 3.1% of all cancer cases [1]. The burden is considerably higher in men than in women, with incidence and mortality rates nearly four times higher among males than among females, making BC the sixth most common cancer and the ninth leading cause of cancer death in men [1].

Geographically, Southern Europe, particularly Spain, has the highest incidence rate in men, while the Netherlands has the highest rate in women. Conversely, the lowest rates are found in Middle Africa and South-Central Asia [1]. Additionally, the relatively intensive surveillance strategies and expensive treatment costs contribute to BC having the highest lifetime treatment costs per patient of all cancers [2, 3]. The heavy burden of BC is not only due to its high prevalence and the aging global population, but also to the high recurrence rate of non-muscle invasive BC, which accounts for around 70% of newly detected cases [4–6].

The occurrence of BC is largely attributed to its risk factors, such as tobacco smoking, occupational factors (e.g., exposure to aromatic amines), and infection with Schistosoma haematobium [1]. Given the bladder’s role as an excretory organ, dietary factors have also been a focus of research for decades [7]. However, the Third Expert Report from the World Cancer Research Fund (WCRF) recently concluded that the evidence on the impact of diet on BC risk is still rather limited [8]. While most of the WCRF’s research focused on single food items (e.g., milk, fruits, and vegetables) and nutrients (e.g., carbohydrates, proteins, and vitamins), assessing overall dietary patterns (DPs) offers a more comprehensive approach due to the complex interactions between various dietary components [9]. Previous observational studies and meta-analyses suggest that the Mediterranean diet may reduce BC risk, while the Western diet is associated with a higher risk [10–12]. Since here is no official dietary pattern recommendations for BC prevention, our study aims to add information in this field by further exploring the relationship between DPs and BC risk.

An additional challenge in studying diet and BC risk is the potential for reverse causation. Dietary habits often change after a disease diagnosis, complicating the interpretation of associations in retrospective observational studies. Moreover, observational studies are susceptible to bias from unmeasured and residual confounders [13]. Mendelian Randomization (MR) is a method used to infer causal relationships by leveraging genetic variants as instrumental variables (IV), allowing for more robust causal inference compared to traditional observational studies [13]. In a population, genetic variants’ random distribution is like that in a randomized controlled trial (RCT), the gold standard for evaluating causal relations. We can compare outcome measures of genetic variant carriers and non-carriers [14]. Genetic variants are fixed at conception and unaffected by outcome development or external factors, so they’re assumed independent of confounders and reverse causation [15]. Also, in the genomic era, genotypes are less likely to have measurement error. Thus, using genetic variants as instrumental variables in MR analyses is a promising way to determine exposure-outcome causality and control biases [15].

Recent genome-wide association studies (GWAS) have indicated that dietary habits are heritable traits with an average heritability of 18%, making them suitable for MR analysis [16–18]. While several published MR have linked specific foods (e.g., fruits, beef, instant coffee and cereals) to the risk of various diseases including BC [19–22], there is no MR study investigating the impact of overall dietary patterns (DPs) on BC risk. To address this gap, our study aims to determine the causal relationship between diet and bladder cancer risk using a two-sample MR approach.

## Methods

### MR design

Two sample Mendelian randomization (TSMR) as one type of MR was employed in the current study, leveraging genetic variants as instrumental variables to estimate causal effects by combining summarize statistics from separate GWAS studies for exposure (diet) and outcome (BC risk), thereby avoiding sample-specific biases and enhancing statistical power [23]. The causal effect of the exposure on the outcome is estimated by combining the two sets of associations. This involves calculating the ratio of the genetic association with the exposure and the genetic association with the outcome for each genetic variant, and then aggregating these ratios across all genetic variants by several methods.

Three core assumptions for instrumental variables (IV) are required: (1) the genetic variant(s) being used as an instrument is associated with the exposure (the relevance assumption); (2) the genetic instruments are not associated with any confounders of the exposure-outcome relationship (the independence assumption); and (3) there is no independent pathway between the genetic variant(s) and outcome other than through the exposure (the exclusion restriction assumption).

This study is reported as per the Strengthening the Reporting of Observational Studies in Epidemiology Using Mendelian Randomization: The STROBE-MR Statement (https://www.strobe-mr.org/) [24].

### Instrument variable selection

To fulill the relevance assumption, genetic IVs, specifically single nucleotide polymorphisms (SNPs), were selected for each exposure at a genome-wide significance threshold (p < 5 × 10^−8^) to minimize the risk of false positives. A clumping procedure was used to ensure independence among SNPs by retaining only the most significant SNP in each region (r^2^ < 0.001, distance = 10Mb), avoiding high linkage disequilibrium (LD) [25]. SNPs with a minor allele frequency (MAF) of less than 0.01 were excluded. The proportion of variance explained by the SNPs (R^2^), was calculated to quantify their relevance, and SNP strength was evaluated using the F statistic, with weak instruments (F < 10) excluded from analysis.

### Exposure data source

Diet was identified as the primary exposure in this study, focusing on 14 specific food/beverage items and four reported DPs. All GWAS data on these exposures were derived from the UK Biobank (UKB) project, a large prospective cohort study involving over 500,000 participants aged 40–69 years at baseline between 2006 and 2010 in the United Kingdom [26]. The 14 food/beverage items included processed meat, red meat (pork, beef, lamb), milk, cheese, butter, whole grains, oily fish, fruits, vegetables, green tea, coffee, and alcohol.These items were selected due to their inconclusive effects on BC risk and underlying biological mechanisms, such as the pro- and anti-inflammatory properties of the diet [27–29]. The GWAS summary-level data for the intake of these food items were obtained from a public database (https://gwas.mrcieu.ac.uk/datasets) and analyzed using the MRC-IEU UKB GWAS pipeline [30]. Sample sizes for each food item ranged from 64,949 for green tea to 462,346 for alcohol.

Among the four DPs, a healthy dietary pattern (PC1) was developed based on single food intake from food frequency questionnaire data of 449,210 participants in the UKB [31]. The summarized data of PC1 were obtained from the GWAS Catalog website (https://www.ebi.ac.uk/gwas/studies/GCST90133006). PC1 was found to be the most heritable and represented a prudent diet. It had a positive association with the increased intake of wholemeal/wholegrain bread, fruits and vegetables, oily fish, and water, while showing a negative association with the increased intake of white bread, butter and oil spreads, processed meat, and higher-fat milk [31].

Three additional DPs were identified in a GWAS study, which was based on the premise that food liking is highly heritable [32]. The summarized data for these patterns were collected from the GWAS Catalog website (https://www.ebi.ac.uk/gwas/publications/35585065). This study analyzed food liking in 161,625 UKB participants, using a 9-point scale to assess preferences for 139 specific foods [32]. Food liking patterns from GWAS consisted of three dimensions: “Highly-palatable dietary pattern” (HPDP), “Acquired dietary pattern” (ADP), and “Low-caloric dietary pattern”(LCDP). The HPDP included energy-rich and widely enjoyed foods like desserts, meat, and savory foods. The LCDP mainly comprised low-caloric foods, such as vegetables, fruits, and whole grains. The ADP included foods generally liked later in life, such as unsweetened coffee, alcohol, cheese, and strong-tasting vegetables [32].

### Outcome data source

The publicly available summary-level data on BC were obtained from the GWAS Catalog website (https://www.ebi.ac.uk/gwas/studies/GCST90041857), including 2264 BC cases and 454,084 controls of European ancestry from the UKB [33]. In this study, a new generalized linear mixed model (GLMM)-based tool, fastGWA - GLMM, was developed to analyze the UKB data of 456,348 individuals, 11,842,647 variants, and 2,989 binary traits. Through this analysis, 259 rare variants associated with 75 traits were identified. All analyses were adjusted for age, age^2^, sex, age*sex, age^2^*sex and the top 20 genetic principal components. Diagnostic data were based on ICD-10 codes and drawn from a combination of hospital records, cancer registries, and verified self-reports.

### Other variables

The associations between selected SNPs and confounder were tested to examine the independent assumption. Smoking is considered as an established risk factor for BC and frequently adjusted in the diet-BC risk study [8]. The overall GWAS data including the information of all SNPs and traits were exported from the GWAS catalog website. Then the significance of the associations between selected SNPs and smoking-releted traits were checked (p < 5 × 10^−8^).

### Statistical analyses

To investigate the causal relationship between various dietary habits and bladder cancer risk, Two-Sample Mendelian Randomization (TSMR) was conducted using summary statistics of genetic variants for both dietary habit and BC risk. The data were harmonized by aligning effect alleles and excluding ambiguous palindromic SNPs.

The primary analysis used SNP-specific Wald estimates (the ratio of genetic associations with the outcome and the exposures), and then applied the Inverse-Variance Weighted (IVW) method to analyze them under a multiplicative random effects model, assuming all SNPs are valid instruments [34]. It calculates a weighted average of the causal estimates from individual genetic variants, with weights determined by the inverse of their variances. Consistent results across different subsets of variants or sensitivity analyses using IVW strengthen the evidence for a causal relationship, whereas deviations may indicate violations of MR assumptions, such as pleiotropy [34]. Causal estimates of the diet and BC risk were presented as odds ratios (ORs) with 95% confidence intervals (CIs). All effect sizes are reported as one-standard-deviation changes. Benjamini-Hochberg correction method was applied to control the false discovery rate (FDR) across multiple exposures [35]. This approach allowed us to adjust for multiple testing and ensure that the proportion of false positives was controlled while maintaining statistical power [35].

In sensitivity analysis, the weighted median estimator was utilized to provide a reliable causal estimate even when up to 50% of the genetic instruments were invalid [36]. This method calculates the median of the causal effects weighted by their precision, thereby mitigating the impact of invalid instruments and enhancing the robustness of the results [36].

To examine the exclusion restriction assumption, heterogeneity of independent SNP effects and MR-Egger regression were used to test the horizontal pleiotropy. Cochran’s Q statistic was calculated to evaluate the degree of heterogeneity, with significance being indicated by a P value less than 0.05 [37]. MR-Egger regression was applied to detect and account for directional pleiotropy. By regressing the genetic associations with the outcome against those with the exposure, the intercept term was tested to identify pleiotropy. A non-zero intercept indicates the presence of pleiotropy. The slope estimate from MR-Egger was used as an alternative and robust causal effect estimate when pleiotropy was suspected [38]. MR-PRESSO (Mendelian Randomization Pleiotropy RESidual Sum and Outlier) was employed to identify and remove horizontal pleiotropic outliers that could distort the causal estimate. After detecting and excluding outliers, the causal effect was re-estimated to ensure results were not driven by spurious associations, thus improving the accuracy of the causal inference [39]. A leave-one-out method was conducted using IVW model for sensitivity analysis to further test the robustness of the causal inference. Then the SNPs corraleted with potential confounder (smoking) were excluded from the main analysis to see whether these SNPs affect the results.

All analyses were conducted using software R version 4.4.1 with packages “TwoSampleMR” and “MRPRESSO”.

## Results

**Figure 1:**
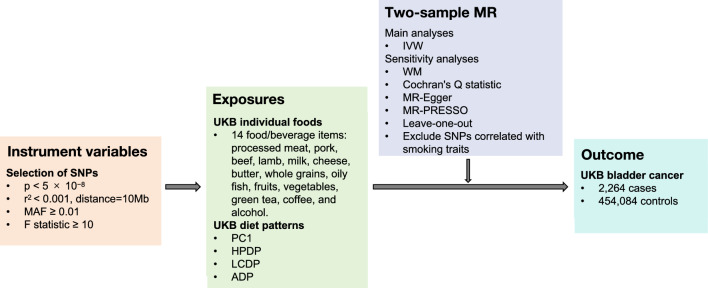
Flow chart of the study design. *ADP* acquired dietary pattern, *HPDP* highly palatable dietary pattern, *IVW* inverse-variance weighted, *LCDP* low-caloric dietary pattern, *MAF* minor allele frequency, *PC1* principal component-derived dietary pattern 1, *WM* weighted median

Selection of data and variables and methods used are shown in the flowchart** (**Figure 1**).**

### Descriptive characteristics of instrumental variables

The summarized data of SNPs for both the exposures and the outcome were extracted from UKB, resulting in a high similarity in the genetic variant-exposure associations between the exposure and outcome samples, as well as a substantial overlap between the two. The details of the SNPs for the 14 food/beverage items and four dietary patterns (DPs), including the number of SNPs, rsIDs, beta, standard error and p-value of SNPs on exposures and the outcome, R^2^, and F-statistics, were presented in Supplementary Table 1.

### Associations between diet and bladder cancer risk

A total of 2264 bladder cancer cases and 454,084 controls were included. The inverse-variance weighted (IVW) analyses (Table 1) showed that adherence to PC1 (high in whole grains, fruits, and vegetables) was significantly associated with a lower risk of bladder cancer (OR: 0.735, 95% CI 0.609–0.888, p = 0.018). In contrast, the HPDP, characterized by processed and high-calorie foods, was associated with an increased risk of bladder cancer (OR: 2.809, 95% CI 1.431–5.515, p = 0.027). However, no significant associations were found between the intake of the 14 individual food and beverage items, or adherence to the LCDP and the ADP, and the risk of BC (Table 1).

**Table 1:** The effect of genetically predicted diet on bladder cancer risk

Foods	Methods	Number of SNPs	OR	95% CI	P-values	Adjusted P-values^a^
Processed meat						
	WM	22	1.306	0.324–5.264	0.708	0.966
	IVW	22	0.915	0.340–2.465	0.861	0.935
Beef						
	WM	15	0.732	0.104–5.13	0.753	0.966
	IVW	15	0.942	0.233–3.807	0.933	0.935
Pork						
	WM	13	0.883	0.079–9.825	0.919	0.973
	IVW	13	0.522	0.084–3.254	0.486	0.935
Lamb						
	WM	31	1.568	0.29–8.48	0.601	0.957
	IVW	31	1.184	0.374–3.747	0.774	0.935
Milk						
	WM	3	0.007	0–748.705	0.401	0.957
	IVW	3	0.077	0–1112.559	0.600	0.935
Cheese						
	WM	63	1.418	0.639–3.149	0.391	0.957
	IVW	63	1.063	0.592–1.912	0.837	0.935
Butter						
	WM	8	0.188	0.004–8.672	0.393	0.957
	IVW	8	0.239	0.015–3.913	0.316	0.935
Whole grains (bread)						
	WM	15	0.156	0.009–2.801	0.208	0.957
	IVW	15	0.135	0.017–1.105	0.062	0.372
Oily fish						
	WM	60	0.808	0.337–1.937	0.633	0.957
	IVW	60	1.115	0.616–2.019	0.720	0.935
Fruit						
	WM	51	1.426	0.325–6.265	0.638	0.957
	IVW	51	0.873	0.322–2.366	0.790	0.935
Vegetable						
	WM	17	0.866	0.104–7.232	0.895	0.973
	IVW	17	0.821	0.165–4.077	0.809	0.935
Green tea						
	WM	13	1.008	0.976–1.042	0.622	0.957
	IVW	13	1.008	0.983–1.033	0.546	0.935
Coffee						
	WM	38	1.013	0.392–2.620	0.979	0.979
	IVW	38	0.972	0.491–1.925	0.935	0.935
Alcohol frequency						
	WM	93	1.322	0.790–2.213	0.288	0.957
	IVW	93	1.252	0.933–1.680	0.134	0.603
PC1 diet pattern						
	WM	100	0.846	0.640–1.119	0.241	0.957
	IVW	100	0.735	0.609–0.888	0.001	0.018
Highly palatable diet pattern						
	WM	17	2.602	1.021–6.633	0.045	0.810
	IVW	17	2.809	1.431–5.515	0.003	0.027
Low caloric diet pattern						
	WM	7	0.899	0.602–1.340	0.601	0.957
	IVW	7	0.775	0.527–1.138	0.193	0.695
Acquired diet pattern						
	WM	35	1.030	0.812–1.307	0.805	0.966
	IVW	35	0.984	0.835–1.160	0.847	0.935

*WM* weighted median, *IVW* inverse variance weighted, *OR* odds ratios, *SNPs* Single Nucleotide Polymorphisms, *CI* confident intervals, *PC1* principal component-derived dietary pattern 1

^a^ The adjusted P-values using Benjamini-Hochberg method

### Robustness and sensitivity analyses

**Figure 2:**
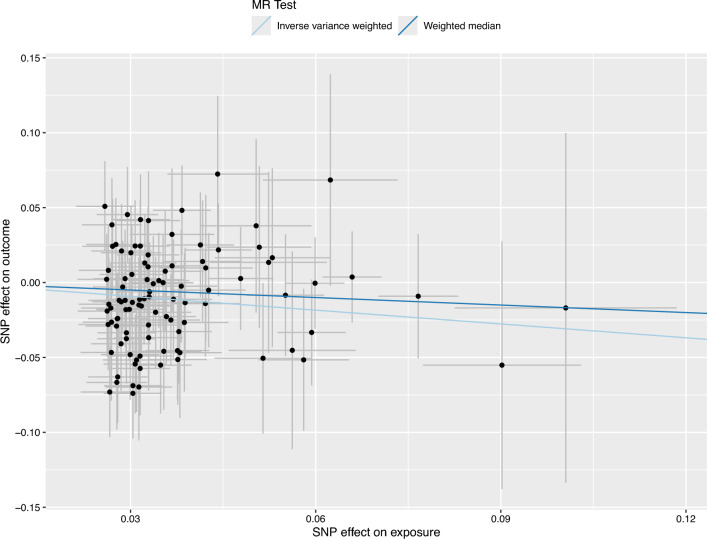
Scatter plot showing the effects of SNPs on principal component-derived dietary pattern 1 versus bladder cancer risk

**Figure 3:**
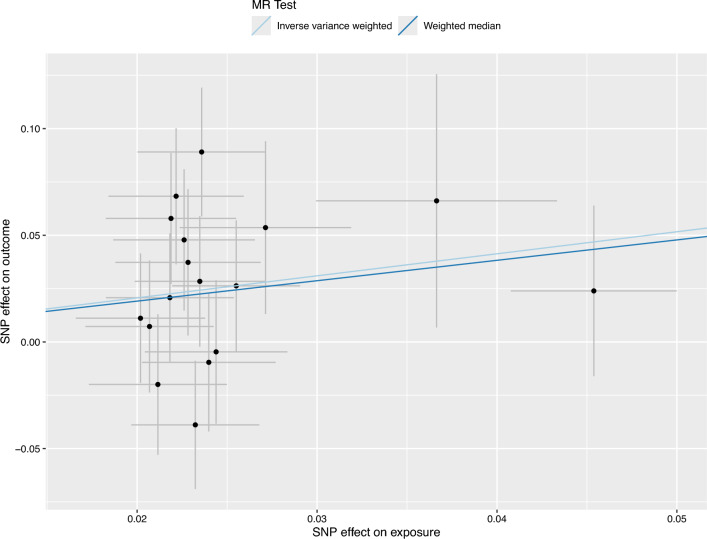
Scatter plot showing the effects of SNPs on highly palatable diet pattern versus bladder cancer risk

Results of MR analyses using the weighted median method confirmed the robustness of the findings for single food/beverage items and DPs (Table 1). Although the association between PC1/HPDP and BC risk was not significant with the weighted median method (Table 1), the direction of the effect remained consistent. The effects of each SNP on exposure and outcome by different MR models are displayed in Figs. 2 and 3 for PC1 and HPDP, respectively.

Additionally, no heterogeneity was detected in the IVW models of PC1 and HPDP, as indicated by Cochran’s Q statistics and its p-value (Table 2, Q statistics: 86.87 and 17.83, p values: 0.803 and 0.335, for PC1 and HPDP, respectively). MR Egger regression revealed no horizontal pleiotropy for the SNPs included in the MR analyses (Table 2, p values for intercept: 0.075 for PC1 and 0.828 for HPDP). Furthermore, no horizontal pleiotropic outliers were identified by MR-PRESSO analyses (Table 2, p values for the global test: 0.809 and 0.350 for PC1 and HPDP, respectively).

**Table 2:** Tests for detecting horizontal pleiotropy

Exposure	Outcome	MR-PRESSO global test		Heterogeneity (IVW)			MR-Egger regression		
		RSS	P-values	Q	df	P-values	Intercept	se	P-values
PC1	BC	93.109	0.809	86.87	99	0.803	−0.023	0.013	0.075
HPDP	BC	20.163	0.350	17.83	16	0.335	0.0094	0.043	0.828

*MR* mendelian randomization, *RSS* residual sum of square, *IVW* inverse variance weight, *df* degree of freedom, *se* standard error, *BC* bladder cancer, *PC1* principal component-derived dietary pattern 1, *HPDP* highly palatable diet pattern

In sensitivity analyses, the associations between PC1, HPDP, and BC risk remained robust under IVW model. Results did not change substantially when excluding one SNP at a time (Supplementary Figures 1 and 2).

The correlations between selected SNPs of the PC1 and the HPDP and smoking traits were examined. For PC1, 6 out of 100 SNPs were significantly associated with smoking traits (p values < 5 × 10^−8^). After excluding the 6 SNPs, PC1 was associated with a decreased BC risk (OR: 0.697, 95% CI 0.574-0.846, p < 0.001), similar to the primary result. Moreover, none of the 17 SNPs of the HPDP was correlated with smoking traits.

## Discussion

In this two-sample Mendelian randomization (MR) study, we utilized GWAS summary statistics to explore the genetic correlations between 14 dietary habits, four DPs, and the risk of BC.

The primary results indicated that there was no significant association between the 14 dietary habits, as well as two of the DPs (the LCDP and the ADP), and the BC risk. However, a higher level of adherence to the “healthy” PC1 was found to decrease the BC risk. Conversely, the HPDP was associated with an increased BC risk. Notably, these findings remained robust across multiple sensitivity analyses. There was no indication of bias caused by pleiotropy or heterogeneity, suggesting the reliability of our results.

The protective effect of PC1 may be attributed to the high content of antioxidants, fiber, and anti-inflammatory compounds in whole grains, fruits, and vegetables, which have been shown to reduce oxidative stress and inflammation—two key mechanisms in carcinogenesis [40–42]. These results are in line with previous observational studies suggesting a protective effect of healthy diets against bladder cancer. For instance, a large-scale cohort study reported a similar association between Mediterranean diet, which is high in whole grains, fruits and vegetables intake and reduced BC risk [10]. The contrasting results between PC1 and the food-liking derived LCDP, both characterized by high intake of whole grains, fruits and vegetables, may be explained by the statistic power. PC1 included more SNPs with a larger sample size, enhancing its ability to detect associations compared to LCDP.

Interestingly, no significant correlations were found between individual components, such as whole grains, fruits, and vegetables, and BC risk in the present study. This supports the concept that dietary patterns exert stronger effects than individual foods through combined mechanisms [9]. For instance fruits and whole grains may have synergistic antioxidant interactions that enhance their protective effects, as shown in previous studies [43, 44]. A healthy DP may also reflect overall healthier lifestyle choices, such as regular exercise and avoiding smoking and alcohol, further contributing to reduced BC risk. Additionally, PC1 has been reported to detect more genetic associations compared to single food items due to its comprehensive inclusion of multiple dietary components [31].

Conversely, higher adherence to the HPDP was associated with an increased risk of BC. This finding aligns with studies that have linked Western dietary patterns, characterized by high intakes of processed meats and high-calorie foods, to higher BC risk [11, 12]. The result might be explained by the high intake of processed foods, which contain pro-inflammatory compounds, carcinogenic additives, as well as excessive amounts of saturated fats and sugar, all of which contribute to cancer development [44–56].

The non-significant result observed for individual processed meat might be explained by the genetic instruments available for this analysis compared to the HPDP. HPDP, based on food-liking, has been shown to have a stronger heritable component, potentially reflecting long-term consumption habits better and thus showing a stronger association with BC risk [32]. The cumulative effect of harmful elements in the HPDP and associated unhealthy lifestyle factors should also be considered.

The present study is the first MR analysis to comprehensively explore the genetic association between diet, characterized as individual food items and dietary patterns, and BC risk using summarized statistics from large-sample GWAS studies. One of the key strengths of this study is the MR study design itself. By using genetic variants as instrumental variables, it effectively minimizes the biases stemming from reverse causation and confounding, which are common issues in traditional observational studies. The use of stringent selection criteria for SNPs and calculation of F-statistics enhances the reliability of the findings by reducing weak instrument bias. Notably, the study’s results are consistent with dietary recommendations by the WCRF for cancer prevention, underscoring the benefits of diets rich in whole grains, fruits, vegetables, and limiting processed foods, red meat, and sugar-sweetened drinks [8].

However, it is essential to acknowledge the limitations of this study. First, MR assumptions may be violated due to undetected horizontal pleiotropy or other pathways through which SNPs might influence the outcome. For example, PC1 has been proved to be genetically correlated with non-diet traits, including physical activity, educational attainment and smoking status, which could confound its association with BC risk [31]. The potential non-linearity of diet and BC risk could also limit the applicability of MR analyses assuming linearity [57]. Another limitation is the use of overlapping samples from the UK Biobank (UKB). Although the large sample size and high-quality data from the UKB can mitigate concerns related to weak instrument bias to some extent, the overlapping nature of the samples may still introduce bias. Regarding the non-significant findings for single food items, they could be attributed to the limited statistical power to detect small effects. Genetic instruments often explain only a small proportion of the phenotypic variance, which may make it difficult to identify associations, especially when the true effects are weak. Lastly, the generalizability of these findings is likely restricted to populations of European ancestry. The results may not be applicable to other ethnic groups, as genetic and environmental factors can vary significantly across different populations.

The findings in this two-sample Mendelian randomization study, revealing the impact of dietary patterns on bladder cancer risk, can guide the public to make healthier dietary choices in line with cancer prevention recommendations. For future research, it’s necessary to address MR assumption violations, explore diet-risk non-linearity, extend studies to other ethnic groups, and improve statistical power for single food items to overcome current limitations. Moreover, conducting other relevant studies employing diverse research methods is justified to verify the current results and explore the underlying mechanisms, thereby enriching the knowledge in the field of bladder cancer prevention.

## Conclusions

Overall, our study found that while the intake of individual food/beverage items are not associated with BC risk, certain dietary patterns like PC1 and HPDP showed significant associations. These findings highlight the importance of considering overall DPs and add insights into the diet recommendations in BC prevention. Future studies should validate these results in diverse populations and explore the specific mechanisms through which dietary components interact with genetic and environmental factors to influence BC risk.

## Data availability

Summarized statistics of GWAS studies used in the study were publicly available as described before. The data sets generated during and/or analyzed during the current study are available from the corresponding author upon reasonable request.

## Supplementary Information

Below is the link to the electronic supplementary material.

Supplementary file1 (PDF 312 KB)

## Abbreviations

ADP: acquired dietary pattern
BC: bladder cancer
CI: confidence interval
DPs: dietary patterns
GWAS: genome-wide association studies
HPDP: Highly Palatable Dietary Pattern
IARC: International Agency for Research on Cancer
ICD-10: the 10th revision of the International Classification of Diseases
IVs: instrumental variables; IVW: inverse-variance weighted
LCDP: low-caloric dietary pattern
LD: linkage disequilibrium
MAF: minor allele frequency
MR: mendelian randomization
OR: odds ratio
PC1: Principal Component-derived dietary pattern 1
SNPs: single nucleotide polymorphisms
TSMR: two-sample mendelian randomization
UKB: UK biabank
WCRF: World Cancer Research Fund
WM: weighted median
