# Supplementary material for: The association between diet and bladder cancer risk: a two-sample mendelian randomization
Source: Eur J Nutr. 2025 Jun 18;64(5):223. doi: 10.1007/s00394-025-03743-5 (PMC12176995; doi:10.1007/s00394-025-03743-5)
Supplement: Supplementary file 1 — Supplementary file1 (PDF 312 KB) [file 394_2025_3743_MOESM1_ESM.pdf]

Yanan Dai et al. The association between diet and bladder cancer risk: a two-sample mendelian randomization

Supplementary Materials

Supplementary Table 1 Summary statistics for the associations of the diet-associated SNPs with the exposures and bladder cancer in UK Biobank

Supplementary Figure 1 Leave-one-out analysis of the effect of PC1 on BC risk

Supplementary Figure 2 Leave-one-out analysis of the effect of HPDP on BC risk

Abbreviations: SNPs, Single Nucleotide Polymorphisms; BC, bladder cancer; PC1, principal component-derived dietary pattern 1; HPDP, highly palatable diet pattern.

Supplementary Table 1 Summary statistics for the associations of the diet-associated SNPs with the exposures and bladder cancer in UK Biobank

NO.	SNP	Sample size	Effect allele	Other allele	EAF	Exposure			Outcome			R^2	F statistic
						Beta	SE	pval	Beta	SE	pval		
	Processed meat	461981											
1	rs10454812		C	A	0.1030	-0.0200	0.0034	6.70E-09	-0.1011	0.0494	0.0406	7.28E-05	33.63
2	rs11032380		T	A	0.3334	-0.0133	0.0022	2.10E-09	0.0113	0.0319	0.7243	7.77E-05	35.92
3	rs11887120		T	C	0.3977	0.0120	0.0022	3.10E-08	-0.0355	0.0312	0.2564	6.64E-05	30.66
4	rs11894162		T	C	0.5475	0.0120	0.0021	1.10E-08	-0.0058	0.0302	0.8483	7.08E-05	32.70
5	rs1422192		A	G	0.1581	0.0170	0.0029	3.40E-09	-0.0621	0.0411	0.1309	7.56E-05	34.92
6	rs2029401		G	A	0.5862	0.0146	0.0021	6.30E-12	0.0124	0.0305	0.6834	1.02E-04	47.22
7	rs203319		T	C	0.2046	-0.0164	0.0026	2.80E-10	-0.0391	0.0374	0.2962	8.62E-05	39.84
8	rs2873054		C	A	0.3533	0.0140	0.0022	1.60E-10	-0.0425	0.0313	0.1741	8.84E-05	40.85
9	rs34241936		G	A	0.0374	0.0328	0.0058	1.10E-08	0.0122	0.0876	0.8890	7.05E-05	32.57
10	rs3762621		T	C	0.1835	-0.0150	0.0027	3.60E-08	-0.0038	0.0391	0.9235	6.58E-05	30.38
11	rs4077924		C	T	0.7019	0.0125	0.0023	4.50E-08	0.0037	0.0327	0.9111	6.48E-05	29.93
12	rs4240672		A	G	0.4940	0.0171	0.0021	3.00E-16	0.0328	0.0299	0.2730	1.45E-04	66.84
13	rs4778053		G	C	0.8438	0.0165	0.0029	1.30E-08	-0.0585	0.0416	0.1602	6.98E-05	32.27
14	rs6010651		C	A	0.3794	-0.0124	0.0022	1.10E-08	-0.0269	0.0311	0.3872	7.09E-05	32.75
15	rs6484504		C	T	0.7246	0.0155	0.0023	4.40E-11	-0.0177	0.0337	0.5995	9.40E-05	43.43
16	rs6765179		A	G	0.3100	-0.0128	0.0023	1.80E-08	-0.0014	0.0325	0.9644	6.86E-05	31.71
17	rs6786550		C	T	0.6350	0.0122	0.0022	2.10E-08	-0.0184	0.0311	0.5543	6.80E-05	31.42
18	rs6961970		A	C	0.2447	-0.0140	0.0024	9.50E-09	0.0180	0.0349	0.6056	7.13E-05	32.95

19	rs7531118		C	T	0.5311	-0.0141	0.0021	2.80E-11	-0.0090	0.0304	0.7686	9.60E-05	44.33
20	rs77165542		T	C	0.0355	0.0339	0.0057	3.30E-09	0.0551	0.0826	0.5047	7.58E-05	35.02
21	rs8096167		C	T	0.1926	-0.0146	0.0027	4.70E-08	0.0576	0.0383	0.1331	6.46E-05	29.86
22	rs9809856		G	A	0.4759	0.0133	0.0021	2.50E-10	0.0232	0.0301	0.4418	8.67E-05	40.06
	Beef	461053											
1	rs10789340		G	A	0.6267	-0.0138	0.0018	6.80E-15	0.0325	0.0311	0.2966	1.32E-04	60.65
2	rs10959890		C	T	0.2123	-0.0127	0.0021	1.50E-09	0.0358	0.0367	0.3301	7.92E-05	36.51
3	rs1105388		T	C	0.3001	-0.0114	0.0019	1.30E-09	0.0246	0.0331	0.4566	7.98E-05	36.78
4	rs11165829		G	C	0.3600	-0.0102	0.0018	9.80E-09	0.0158	0.0311	0.6115	7.13E-05	32.89
5	rs11878917		A	G	0.1097	0.0150	0.0027	4.60E-08	0.0220	0.0484	0.6498	6.48E-05	29.87
6	rs132901		T	C	0.7877	0.0139	0.0021	2.90E-11	0.0357	0.0367	0.3311	9.60E-05	44.27
7	rs1421085		C	T	0.4034	-0.0121	0.0017	3.50E-12	0.0037	0.0305	0.9040	1.05E-04	48.39
8	rs1470610		C	G	0.1962	-0.0122	0.0022	1.50E-08	0.0131	0.0379	0.7286	6.95E-05	32.05
9	rs429358		C	T	0.1542	-0.0149	0.0024	3.60E-10	-0.0091	0.0414	0.8254	8.53E-05	39.32
10	rs4676964		T	C	0.5106	0.0134	0.0017	9.60E-15	0.0734	0.0306	0.0163	1.30E-04	59.97
11	rs62169335		T	C	0.5432	-0.0097	0.0017	2.40E-08	-0.0241	0.0305	0.4301	6.76E-05	31.16
12	rs62396185		C	G	0.2601	-0.0148	0.0020	2.80E-14	0.0047	0.0343	0.8917	1.26E-04	57.89
13	rs7791463		A	G	0.5348	0.0095	0.0017	2.40E-08	0.0046	0.0300	0.8768	6.76E-05	31.16
14	rs784251		T	C	0.4776	-0.0103	0.0017	1.70E-09	0.0120	0.0302	0.6910	7.87E-05	36.28
15	rs79809011		A	G	0.0295	-0.0281	0.0051	3.40E-08	0.1349	0.0888	0.1289	6.60E-05	30.45
	Pork	460162											
1	rs10972033		T	G	0.4564	0.0090	0.0015	1.30E-09	0.0414	0.0300	0.1673	8.01E-05	36.85
2	rs11211124		C	T	0.2306	-0.0100	0.0018	1.40E-08	-0.0106	0.0357	0.7669	7.00E-05	32.19
3	rs12721051		G	C	0.1884	-0.0124	0.0019	5.60E-11	0.0016	0.0383	0.9667	9.33E-05	42.94

4	rs1355171		A	C	0.4888	-0.0110	0.0015	1.00E-13	-0.0057	0.0301	0.8506	1.20E-04	55.30
5	rs2387807		T	C	0.0779	-0.0151	0.0027	4.10E-08	0.1208	0.0563	0.0319	6.55E-05	30.12
6	rs254152		G	C	0.2350	-0.0104	0.0017	2.20E-09	0.0231	0.0355	0.5159	7.78E-05	35.82
7	rs34161520		G	C	0.1604	0.0116	0.0020	9.60E-09	-0.0011	0.0410	0.9793	7.15E-05	32.91
8	rs36124222		C	T	0.4332	0.0084	0.0015	2.10E-08	-0.0162	0.0307	0.5984	6.82E-05	31.40
9	rs3964074		C	T	0.5469	-0.0090	0.0015	1.60E-09	0.0010	0.0302	0.9730	7.91E-05	36.41
10	rs4146837		T	C	0.4556	0.0088	0.0015	4.00E-09	-0.0147	0.0306	0.6304	7.52E-05	34.62
11	rs7641973		A	G	0.3534	0.0084	0.0015	4.20E-08	-0.0407	0.0312	0.1919	6.53E-05	30.04
12	rs9379832		G	A	0.2555	-0.0115	0.0017	1.80E-11	0.0154	0.0349	0.6598	9.82E-05	45.20
13	rs9973426		G	A	0.1766	0.0111	0.0019	1.00E-08	0.0374	0.0395	0.3429	7.12E-05	32.76
	Lamb	460006											
1	rs11090045		A	G	0.3071	-0.0107	0.0016	3.00E-11	-0.0316	0.0332	0.3414	9.60E-05	44.16
2	rs11743441		T	G	0.5743	-0.0088	0.0015	2.70E-09	0.0230	0.0306	0.4516	7.70E-05	35.41
3	rs12634740		G	T	0.2521	-0.0101	0.0017	2.80E-09	0.0025	0.0347	0.9434	7.68E-05	35.32
4	rs136548		T	C	0.3767	0.0095	0.0015	2.90E-10	-0.0469	0.0310	0.1299	8.64E-05	39.73
5	rs139237013		A	G	0.0577	0.0189	0.0031	1.80E-09	-0.0533	0.0643	0.4074	7.87E-05	36.22
6	rs1556147		T	A	0.6716	0.0091	0.0016	5.30E-09	0.0020	0.0319	0.9489	7.41E-05	34.08
7	rs16891982		G	C	0.9721	-0.0243	0.0044	2.70E-08	0.0495	0.0965	0.6083	6.72E-05	30.92
8	rs17270057		C	T	0.1133	0.0127	0.0023	4.30E-08	0.0013	0.0473	0.9778	6.53E-05	30.03
9	rs1958801		G	A	0.2880	-0.0089	0.0016	3.20E-08	-0.0600	0.0332	0.0708	6.65E-05	30.58
10	rs2222760		A	G	0.2809	-0.0091	0.0016	2.80E-08	0.0327	0.0336	0.3305	6.70E-05	30.84
11	rs2678900		G	T	0.4279	0.0101	0.0015	9.90E-12	-0.0167	0.0304	0.5818	1.01E-04	46.35
12	rs2726033		G	A	0.4224	-0.0095	0.0015	1.50E-10	0.0385	0.0303	0.2036	8.93E-05	41.09
13	rs276453		C	A	0.4884	-0.0142	0.0015	2.90E-22	-0.0153	0.0301	0.6115	2.05E-04	94.15

14	rs2926119		A	C	0.5694	0.0081	0.0015	4.40E-08	0.0029	0.0304	0.9239	6.51E-05	29.96
15	rs3105056		C	T	0.7327	-0.0116	0.0016	1.80E-12	-0.0232	0.0337	0.4913	1.08E-04	49.72
16	rs35797675		G	T	0.2159	-0.0108	0.0018	1.40E-09	0.0072	0.0371	0.8453	7.96E-05	36.60
17	rs3964074		C	T	0.5470	-0.0081	0.0015	3.20E-08	0.0010	0.0302	0.9730	6.65E-05	30.58
18	rs4272399		A	C	0.3215	-0.0092	0.0016	4.50E-09	0.0229	0.0324	0.4792	7.48E-05	34.41
19	rs429358		C	T	0.1542	-0.0182	0.0020	2.70E-19	-0.0091	0.0414	0.8254	1.75E-04	80.61
20	rs4489752		T	G	0.8360	0.0138	0.0020	2.80E-12	0.0439	0.0405	0.2789	1.06E-04	48.86
21	rs55813438		A	G	0.7632	-0.0114	0.0017	4.70E-11	-0.0579	0.0358	0.1057	9.41E-05	43.30
22	rs56394517		G	A	0.0958	-0.0138	0.0025	3.20E-08	-0.0334	0.0513	0.5155	6.65E-05	30.57
23	rs62106258		C	T	0.0486	0.0216	0.0034	2.00E-10	0.0725	0.0696	0.2972	8.79E-05	40.44
24	rs62398404		T	C	0.1272	0.0129	0.0022	4.00E-09	0.0818	0.0447	0.0672	7.53E-05	34.64
25	rs6581296		G	C	0.7947	0.0100	0.0018	4.00E-08	0.0333	0.0375	0.3752	6.56E-05	30.17
26	rs660880		A	G	0.5128	-0.0090	0.0015	6.80E-10	0.0654	0.0300	0.0292	8.27E-05	38.06
27	rs673696		T	C	0.0810	0.0158	0.0027	3.70E-09	0.0408	0.0549	0.4578	7.56E-05	34.80
28	rs6829572		A	G	0.4567	0.0084	0.0015	1.20E-08	-0.0100	0.0303	0.7408	7.06E-05	32.48
29	rs7447465		C	T	0.6194	0.0096	0.0015	2.00E-10	0.0065	0.0308	0.8333	8.80E-05	40.47
30	rs7550173		T	A	0.6103	-0.0091	0.0015	1.30E-09	0.0544	0.0308	0.0778	8.02E-05	36.88
31	rs994270		G	C	0.2349	0.0098	0.0017	1.40E-08	0.0136	0.0354	0.7013	7.01E-05	32.24
	Milk												
1	rs11605348		A	G	0.3500	-0.0036	0.0006	1.42E-09	0.0269	0.0314	0.3907	1.02E-04	36.65
2	rs12149832		A	G	0.4119	-0.0040	0.0006	6.52E-12	-0.0179	0.0303	0.5552	1.31E-04	47.17
3	rs72783775		A	G	0.0860	-0.0057	0.0010	3.01E-08	0.0434	0.0537	0.4182	8.51E-05	30.70
	Cheese	451486											
1	rs1073242		A	G	0.5538	0.0157	0.0023	6.70E-12	-0.0057	0.0312	0.8558	1.04E-04	47.11

2	rs10896050	T	G	0.1932	-0.0185	0.0028	7.20E-11	-0.0517	0.0381	0.1740	9.40E-05	42.46
3	rs10938397	G	A	0.4345	-0.0127	0.0023	1.80E-08	-0.0148	0.0303	0.6262	7.02E-05	31.68
4	rs113367286	T	C	0.2785	0.0152	0.0025	1.30E-09	-0.0819	0.0335	0.0146	8.17E-05	36.87
5	rs11620149	C	T	0.1433	-0.0177	0.0032	3.60E-08	-0.0170	0.0432	0.6931	6.72E-05	30.35
6	rs11649653	G	C	0.3821	0.0138	0.0023	1.50E-09	0.0619	0.0307	0.0439	8.09E-05	36.52
7	rs12296440	A	G	0.1697	0.0188	0.0030	2.80E-10	-0.0066	0.0401	0.8698	8.81E-05	39.78
8	rs12447542	A	G	0.1256	0.0197	0.0034	6.80E-09	0.0692	0.0461	0.1333	7.44E-05	33.60
9	rs12475594	G	A	0.1785	0.0160	0.0029	4.40E-08	-0.0275	0.0394	0.4847	6.63E-05	29.95
10	rs12672200	A	G	0.3258	-0.0138	0.0024	9.00E-09	-0.0146	0.0322	0.6498	7.32E-05	33.04
11	rs12786959	T	A	0.1963	-0.0161	0.0028	1.20E-08	-0.0052	0.0378	0.8900	7.19E-05	32.48
12	rs1291145	C	T	0.6858	-0.0202	0.0024	4.40E-17	-0.0925	0.0324	0.0042	1.56E-04	70.58
13	rs12951057	G	C	0.1656	-0.0212	0.0030	3.60E-12	0.0244	0.0411	0.5524	1.07E-04	48.31
14	rs13107325	T	C	0.0747	-0.0292	0.0043	7.00E-12	0.0561	0.0568	0.3229	1.04E-04	47.03
15	rs1434511	T	C	0.4553	0.0130	0.0023	9.50E-09	-0.0435	0.0304	0.1527	7.30E-05	32.95
16	rs1514755	G	A	0.2396	0.0164	0.0026	3.90E-10	-0.0564	0.0351	0.1079	8.67E-05	39.17
17	rs17115145	T	C	0.4013	-0.0129	0.0023	1.80E-08	0.0103	0.0308	0.7378	7.01E-05	31.65
18	rs1806771	G	T	0.0879	-0.0221	0.0040	4.10E-08	-0.0405	0.0553	0.4643	6.67E-05	30.13
19	rs1931805	C	T	0.5001	0.0126	0.0022	1.60E-08	0.0142	0.0300	0.6362	7.07E-05	31.94
20	rs2339928	A	G	0.7041	0.0149	0.0024	1.20E-09	-0.0305	0.0327	0.3516	8.18E-05	36.93
21	rs2352974	T	C	0.4898	-0.0145	0.0022	1.00E-10	-0.0239	0.0302	0.4275	9.25E-05	41.78
22	rs26579	C	G	0.5862	-0.0128	0.0023	2.40E-08	0.0040	0.0312	0.8969	6.89E-05	31.13
23	rs2802530	A	G	0.8765	0.0186	0.0034	4.20E-08	-0.0482	0.0455	0.2894	6.66E-05	30.07
24	rs2854175	A	C	0.2575	0.0170	0.0026	3.70E-11	-0.0135	0.0345	0.6967	9.69E-05	43.77
25	rs2960578	G	T	0.4963	0.0170	0.0022	2.60E-14	0.0063	0.0299	0.8329	1.28E-04	58.00

26	rs34198643	T	C	0.2242	-0.0167	0.0027	4.50E-10	0.0421	0.0360	0.2414	8.61E-05	38.87
27	rs35270670	G	A	0.2180	0.0164	0.0027	1.50E-09	0.0375	0.0364	0.3030	8.09E-05	36.54
28	rs3911016	G	T	0.1209	0.0214	0.0034	5.30E-10	-0.0467	0.0463	0.3130	8.54E-05	38.55
29	rs4296548	G	T	0.6096	0.0130	0.0023	1.20E-08	0.0116	0.0307	0.7049	7.18E-05	32.41
30	rs4503172	T	C	0.6083	0.0130	0.0023	1.60E-08	0.0410	0.0307	0.1827	7.07E-05	31.94
31	rs4681981	A	C	0.4691	-0.0124	0.0022	2.90E-08	-0.0262	0.0300	0.3834	6.82E-05	30.79
32	rs4692708	C	A	0.2527	0.0147	0.0026	1.30E-08	-0.0052	0.0347	0.8805	7.17E-05	32.37
33	rs4776970	T	A	0.3580	0.0154	0.0023	3.50E-11	0.0260	0.0312	0.4039	9.71E-05	43.85
34	rs4860341	C	T	0.9287	0.0244	0.0044	2.20E-08	-0.0500	0.0586	0.3936	6.94E-05	31.35
35	rs504675	T	C	0.3526	0.0274	0.0023	1.00E-31	0.0376	0.0314	0.2317	3.04E-04	137.32
36	rs524468	G	A	0.2606	-0.0142	0.0026	2.40E-08	-0.0002	0.0343	0.9964	6.90E-05	31.15
37	rs531358	T	C	0.6498	0.0132	0.0023	1.80E-08	0.0152	0.0313	0.6285	7.03E-05	31.73
38	rs6126641	A	G	0.3362	0.0132	0.0024	3.30E-08	0.0373	0.0325	0.2514	6.75E-05	30.50
39	rs61734410	T	C	0.2552	0.0167	0.0026	2.20E-10	-0.0329	0.0360	0.3609	8.93E-05	40.32
40	rs61953351	T	G	0.2504	0.0146	0.0026	1.50E-08	-0.0498	0.0346	0.1498	7.09E-05	31.99
41	rs62034322	A	G	0.3798	-0.0139	0.0023	1.40E-09	0.0398	0.0308	0.1967	8.13E-05	36.71
42	rs62236533	A	G	0.1088	0.0248	0.0036	1.10E-11	0.0365	0.0496	0.4613	1.02E-04	46.11
43	rs62245792	A	T	0.1500	-0.0179	0.0032	1.40E-08	-0.0152	0.0427	0.7217	7.12E-05	32.15
44	rs6685323	T	C	0.3093	-0.0132	0.0024	4.80E-08	0.0554	0.0324	0.0879	6.60E-05	29.80
45	rs67238148	T	G	0.2175	0.0165	0.0027	1.10E-09	-0.0091	0.0363	0.8019	8.23E-05	37.15
46	rs6774906	C	A	0.0407	0.0316	0.0057	2.50E-08	-0.0817	0.0760	0.2829	6.89E-05	31.09
47	rs6873324	C	A	0.4258	-0.0125	0.0023	3.90E-08	0.0380	0.0306	0.2151	6.69E-05	30.19
48	rs7012814	A	G	0.4740	-0.0185	0.0023	2.10E-16	0.0371	0.0303	0.2199	1.49E-04	67.49
49	rs71386942	A	C	0.2689	0.0145	0.0025	9.90E-09	-0.0078	0.0337	0.8176	7.28E-05	32.85

50	rs72970243		A	G	0.1204	0.0222	0.0034	6.70E-11	-0.0208	0.0465	0.6541	9.43E-05	42.60
51	rs7298331		C	A	0.6045	-0.0132	0.0023	1.10E-08	0.0226	0.0311	0.4672	7.23E-05	32.65
52	rs73024305		C	G	0.0548	0.0325	0.0049	4.00E-11	0.1165	0.0661	0.0783	9.66E-05	43.61
53	rs73096946		C	T	0.1574	-0.0206	0.0031	1.90E-11	-0.0297	0.0412	0.4716	9.99E-05	45.09
54	rs73335955		C	T	0.0533	0.0278	0.0050	2.40E-08	0.0605	0.0670	0.3665	6.89E-05	31.12
55	rs7386207		T	C	0.5635	-0.0125	0.0023	3.60E-08	-0.0510	0.0305	0.0943	6.72E-05	30.33
56	rs77742462		G	A	0.0205	-0.0475	0.0083	9.80E-09	0.2268	0.1234	0.0662	7.28E-05	32.88
57	rs78876700		A	G	0.1374	0.0181	0.0033	3.40E-08	-0.0997	0.0446	0.0253	6.75E-05	30.47
58	rs79184944		A	T	0.1343	0.0196	0.0033	2.40E-09	0.0485	0.0444	0.2741	7.89E-05	35.61
59	rs7936836		A	C	0.4175	0.0159	0.0023	2.60E-12	0.0349	0.0305	0.2522	1.08E-04	48.96
60	rs919109		C	G	0.1388	0.0199	0.0032	7.90E-10	0.0122	0.0435	0.7790	8.37E-05	37.79
61	rs9504123		C	A	0.2747	0.0142	0.0025	1.50E-08	0.0163	0.0337	0.6276	7.09E-05	32.03
62	rs9649582		T	A	0.3173	-0.0146	0.0024	1.40E-09	-0.0099	0.0324	0.7592	8.14E-05	36.74
63	rs975303		G	A	0.1813	0.0213	0.0029	2.50E-13	-0.0035	0.0391	0.9281	1.19E-04	53.56
	Butter	360291											
1	rs1065853		T	G	0.0803	0.0211	0.0021	4.15E-24	-0.0452	0.0551	0.4115	2.85E-04	102.59
2	rs2445304		G	A	0.5028	-0.0070	0.0011	5.42E-10	-0.0307	0.0302	0.3093	1.07E-04	38.52
3	rs4610260		A	G	0.3107	-0.0072	0.0012	3.76E-09	0.0500	0.0324	0.1225	9.64E-05	34.75
4	rs55730499		T	C	0.0814	-0.0127	0.0021	8.71E-10	-0.0165	0.0551	0.7653	1.04E-04	37.60
5	rs624136		G	A	0.4759	-0.0069	0.0011	1.16E-09	0.0306	0.0299	0.3070	1.03E-04	37.04
6	rs6474930		G	A	0.4875	-0.0066	0.0011	5.18E-09	-0.0165	0.0302	0.5850	9.47E-05	34.12
7	rs6973700		G	A	0.2044	-0.0079	0.0014	2.28E-08	0.0375	0.0371	0.3120	8.67E-05	31.24
8	rs72863305		A	T	0.2501	-0.0071	0.0013	4.76E-08	0.0038	0.0347	0.9136	8.27E-05	29.82
	Whole grains	348424											

	(bread type)												
1	rs10168768		T	G	0.5763	-0.0066	0.0012	2.99E-08	0.0118	0.0303	0.6968	8.82E-05	30.72
2	rs11227478		A	G	0.2001	-0.0085	0.0015	7.79E-09	0.0688	0.0378	0.0684	9.56E-05	33.33
3	rs11692083		A	G	0.5618	0.0070	0.0012	4.51E-09	-0.0327	0.0305	0.2844	9.87E-05	34.39
4	rs1183668		G	C	0.3699	0.0069	0.0012	1.33E-08	-0.0409	0.0313	0.1923	9.27E-05	32.29
5	rs12615184		G	C	0.6071	-0.0066	0.0012	3.57E-08	-0.0122	0.0309	0.6939	8.72E-05	30.37
6	rs1421085		C	T	0.4018	0.0100	0.0012	4.60E-17	0.0037	0.0305	0.9040	2.02E-04	70.51
7	rs1788784		G	A	0.6564	0.0072	0.0012	6.98E-09	0.0218	0.0316	0.4905	9.63E-05	33.54
8	rs2852326		A	C	0.3541	0.0070	0.0012	1.37E-08	-0.0516	0.0316	0.1023	9.25E-05	32.23
9	rs3096698		C	T	0.6488	-0.0075	0.0012	1.04E-09	-0.0026	0.0315	0.9348	1.07E-04	37.25
10	rs35780153		T	C	0.2806	0.0079	0.0013	1.95E-09	-0.0294	0.0339	0.3857	1.03E-04	36.03
11	rs3826209		G	A	0.6232	0.0069	0.0012	1.50E-08	0.0332	0.0314	0.2907	9.20E-05	32.06
12	rs6882046		G	A	0.2647	0.0080	0.0013	2.71E-09	-0.0328	0.0346	0.3431	1.02E-04	35.38
13	rs6978112		T	C	0.4117	-0.0075	0.0012	2.55E-10	0.0141	0.0306	0.6442	1.15E-04	39.99
14	rs7742854		T	C	0.3947	0.0094	0.0012	7.14E-15	-0.0281	0.0308	0.3614	1.74E-04	60.57
15	rs889925		C	T	0.3841	-0.0070	0.0012	5.86E-09	0.0100	0.0310	0.7479	9.72E-05	33.88
	Oily fish	460443											
1	rs10061973		T	G	0.5139	-0.0109	0.0019	1.50E-08	0.0054	0.0300	0.8562	6.97E-05	32.08
2	rs10076975		C	T	0.3814	0.0112	0.0020	1.10E-08	-0.0167	0.0307	0.5866	7.09E-05	32.64
3	rs10510554		C	T	0.5693	0.0110	0.0019	1.20E-08	0.0333	0.0304	0.2740	7.07E-05	32.53
4	rs10513136		A	G	0.0654	-0.0233	0.0039	1.60E-09	-0.0843	0.0607	0.1648	7.92E-05	36.45
5	rs10828250		G	C	0.3093	-0.0201	0.0021	2.60E-22	0.0367	0.0326	0.2607	2.05E-04	94.41
6	rs114497213		T	G	0.0548	0.0273	0.0042	1.10E-10	0.0382	0.0670	0.5690	9.03E-05	41.60
7	rs11767283		G	A	0.2217	0.0177	0.0023	2.50E-14	0.0106	0.0366	0.7727	1.26E-04	58.06

8	rs11859365	C	A	0.2538	0.0226	0.0022	9.40E-25	-0.0263	0.0345	0.4456	2.29E-04	105.53
9	rs1201289	G	T	0.3945	-0.0107	0.0020	4.40E-08	-0.0240	0.0307	0.4353	6.51E-05	29.97
10	rs12663865	A	G	0.7582	0.0128	0.0022	1.10E-08	0.0064	0.0350	0.8555	7.11E-05	32.73
11	rs12855717	T	C	0.5268	-0.0122	0.0019	2.00E-10	0.0077	0.0300	0.7965	8.79E-05	40.46
12	rs12896749	C	G	0.3847	-0.0110	0.0020	2.50E-08	-0.0248	0.0307	0.4190	6.75E-05	31.10
13	rs12983532	T	C	0.2511	-0.0134	0.0022	2.00E-09	-0.0588	0.0355	0.0971	7.81E-05	35.94
14	rs13070166	A	T	0.2286	0.0142	0.0023	4.40E-10	0.0321	0.0357	0.3685	8.45E-05	38.91
15	rs1361016	G	T	0.8449	0.0150	0.0027	1.70E-08	0.0178	0.0418	0.6700	6.90E-05	31.78
16	rs1421085	C	T	0.4034	0.0185	0.0019	2.50E-21	0.0037	0.0305	0.9040	1.95E-04	89.94
17	rs16891727	A	C	0.1298	-0.0237	0.0028	6.80E-17	0.0047	0.0444	0.9150	1.51E-04	69.74
18	rs17050031	T	C	0.4801	-0.0120	0.0019	3.50E-10	0.0119	0.0302	0.6925	8.55E-05	39.35
19	rs1876245	C	T	0.4315	0.0151	0.0019	5.00E-15	-0.0241	0.0302	0.4251	1.33E-04	61.27
20	rs1951286	G	T	0.6449	-0.0146	0.0020	3.00E-13	-0.0446	0.0312	0.1534	1.16E-04	53.20
21	rs2374424	G	A	0.6015	-0.0114	0.0020	4.90E-09	0.0770	0.0307	0.0121	7.43E-05	34.23
22	rs275160	C	T	0.7006	0.0121	0.0021	8.00E-09	0.0340	0.0334	0.3085	7.23E-05	33.27
23	rs2827161	G	T	0.4228	0.0107	0.0019	3.20E-08	0.0126	0.0304	0.6778	6.64E-05	30.57
24	rs28533540	A	G	0.5342	0.0146	0.0019	2.80E-14	-0.0160	0.0303	0.5971	1.26E-04	57.87
25	rs28623270	T	A	0.1487	-0.0178	0.0027	7.30E-11	0.0293	0.0436	0.5011	9.22E-05	42.44
26	rs2952140	T	C	0.4826	-0.0107	0.0019	2.50E-08	0.0383	0.0300	0.2013	6.75E-05	31.09
27	rs303817	G	A	0.7511	0.0136	0.0022	8.00E-10	0.0288	0.0346	0.4056	8.20E-05	37.77
28	rs3124402	G	A	0.7333	-0.0220	0.0022	1.90E-24	-0.0253	0.0337	0.4537	2.26E-04	104.09
29	rs35287743	T	G	0.1159	-0.0282	0.0030	7.00E-21	0.0516	0.0474	0.2766	1.91E-04	87.86
30	rs4002471	T	C	0.5474	-0.0192	0.0019	1.50E-23	0.0268	0.0302	0.3744	2.17E-04	100.00
31	rs4278546	G	A	0.4411	0.0126	0.0019	9.30E-11	0.0117	0.0306	0.7012	9.11E-05	41.97

32	rs4510068	T	G	0.4028	-0.0130	0.0020	4.00E-11	0.0090	0.0311	0.7724	9.47E-05	43.60
33	rs45501495	T	C	0.2360	0.0157	0.0023	3.70E-12	-0.0298	0.0354	0.3994	1.05E-04	48.26
34	rs4869859	C	T	0.4499	0.0140	0.0019	3.10E-13	-0.0308	0.0301	0.3059	1.15E-04	53.13
35	rs4982738	A	G	0.5829	0.0109	0.0020	3.50E-08	0.0388	0.0314	0.2166	6.60E-05	30.41
36	rs510161	G	C	0.3104	-0.0113	0.0021	4.50E-08	-0.0115	0.0324	0.7229	6.50E-05	29.91
37	rs552234	A	G	0.4954	-0.0116	0.0019	1.10E-09	-0.0411	0.0300	0.1697	8.05E-05	37.09
38	rs55930451	T	C	0.1080	-0.0171	0.0031	2.90E-08	0.0182	0.0483	0.7058	6.68E-05	30.74
39	rs55985303	A	G	0.2411	0.0130	0.0022	6.60E-09	-0.0257	0.0351	0.4636	7.31E-05	33.66
40	rs59355765	T	C	0.1601	-0.0163	0.0026	4.70E-10	-0.0218	0.0410	0.5949	8.42E-05	38.78
41	rs6033437	A	C	0.2573	0.0125	0.0022	1.70E-08	-0.0158	0.0351	0.6514	6.91E-05	31.84
42	rs6059844	G	A	0.4951	0.0110	0.0019	9.20E-09	-0.0364	0.0300	0.2247	7.17E-05	33.01
43	rs6089753	T	C	0.5310	-0.0115	0.0019	1.80E-09	-0.0090	0.0300	0.7650	7.86E-05	36.20
44	rs61882686	A	C	0.0852	0.0198	0.0034	8.00E-09	0.0473	0.0538	0.3799	7.22E-05	33.26
45	rs631490	C	G	0.7091	-0.0151	0.0021	6.00E-13	0.0082	0.0329	0.8034	1.13E-04	51.85
46	rs6465487	G	A	0.3998	-0.0124	0.0020	2.70E-10	0.0125	0.0307	0.6842	8.66E-05	39.88
47	rs703987	C	G	0.6150	0.0111	0.0020	1.70E-08	-0.0012	0.0310	0.9697	6.90E-05	31.76
48	rs7243428	G	A	0.2246	-0.0130	0.0023	1.50E-08	0.0151	0.0358	0.6728	6.96E-05	32.06
49	rs7254235	G	A	0.5773	-0.0106	0.0019	4.30E-08	-0.0782	0.0305	0.0103	6.52E-05	30.02
50	rs75887709	G	A	0.1359	-0.0159	0.0028	1.60E-08	-0.0631	0.0440	0.1511	6.94E-05	31.95
51	rs7683782	G	C	0.8334	0.0145	0.0026	1.90E-08	0.0193	0.0405	0.6342	6.87E-05	31.64
52	rs790564	C	A	0.7230	0.0147	0.0021	7.90E-12	-0.0489	0.0338	0.1476	1.02E-04	46.79
53	rs905575	G	C	0.8240	0.0139	0.0025	3.60E-08	-0.0902	0.0397	0.0231	6.59E-05	30.37
54	rs9301837	A	C	0.1433	-0.0157	0.0027	8.10E-09	-0.0497	0.0429	0.2473	7.22E-05	33.24
55	rs9597870	G	T	0.2458	-0.0127	0.0022	1.10E-08	-0.0645	0.0350	0.0658	7.09E-05	32.65

56	rs9606833		C	T	0.2436	0.0170	0.0022	2.70E-14	0.0480	0.0349	0.1690	1.26E-04	57.97
57	rs973526		T	C	0.5133	-0.0115	0.0019	2.50E-09	-0.0084	0.0305	0.7842	7.72E-05	35.56
58	rs9841174		C	T	0.3739	0.0148	0.0020	8.50E-14	-0.0065	0.0310	0.8327	1.21E-04	55.69
59	rs9889161		T	G	0.3579	-0.0133	0.0020	2.80E-11	0.0030	0.0314	0.9241	9.62E-05	44.29
60	rs9958909		G	T	0.1397	0.0158	0.0028	1.40E-08	0.0011	0.0437	0.9804	6.99E-05	32.19
	Fruits	446462											
1	rs10064431		C	T	0.5225	-0.0076	0.0012	6.00E-10	0.0147	0.0300	0.6249	8.58E-05	38.31
2	rs10192394		T	C	0.5288	-0.0077	0.0012	4.50E-10	-0.0243	0.0304	0.4226	8.71E-05	38.88
3	rs10249294		A	G	0.3730	0.0196	0.0013	4.10E-54	0.0254	0.0309	0.4118	5.37E-04	239.94
4	rs10271924		T	C	0.4926	-0.0070	0.0013	2.00E-08	-0.0273	0.0315	0.3862	7.05E-05	31.48
5	rs1051547		C	T	0.5615	-0.0076	0.0012	1.10E-09	-0.0089	0.0303	0.7694	8.34E-05	37.22
6	rs10828266		G	A	0.7156	0.0124	0.0014	8.10E-20	-0.0457	0.0333	0.1700	1.86E-04	83.02
7	rs10838724		T	G	0.3680	0.0090	0.0013	2.10E-12	0.0185	0.0320	0.5636	1.11E-04	49.34
8	rs10840126		G	A	0.3761	-0.0077	0.0013	1.90E-09	-0.0420	0.0321	0.1917	8.07E-05	36.03
9	rs11032362		A	G	0.0910	0.0124	0.0021	5.30E-09	0.0825	0.0521	0.1131	7.63E-05	34.06
10	rs11085749		A	G	0.3871	-0.0077	0.0013	7.10E-10	0.0087	0.0307	0.7764	8.51E-05	37.99
11	rs11248509		T	A	0.3712	0.0073	0.0013	7.40E-09	-0.0202	0.0311	0.5154	7.48E-05	33.41
12	rs11896330		A	G	0.6328	-0.0084	0.0013	3.40E-11	-0.0272	0.0314	0.3869	9.84E-05	43.92
13	rs12044599		G	A	0.2101	0.0094	0.0015	3.70E-10	-0.0215	0.0369	0.5602	8.80E-05	39.27
14	rs12536253		C	G	0.2490	-0.0082	0.0014	8.30E-09	-0.0034	0.0348	0.9213	7.44E-05	33.20
15	rs12641371		T	C	0.4331	0.0079	0.0012	1.40E-10	-0.0129	0.0302	0.6686	9.22E-05	41.15
16	rs12780952		A	G	0.2864	0.0075	0.0014	3.40E-08	-0.0216	0.0333	0.5157	6.83E-05	30.49
17	rs12885598		A	G	0.5967	0.0075	0.0012	1.70E-09	-0.0083	0.0306	0.7861	8.13E-05	36.30
18	rs13072255		C	A	0.4940	0.0090	0.0012	2.10E-13	-0.0350	0.0300	0.2445	1.21E-04	53.87

19	rs1356292	T	C	0.8075	0.0092	0.0016	3.50E-09	0.0542	0.0382	0.1560	7.81E-05	34.89
20	rs1375566	A	G	0.6274	-0.0078	0.0013	6.10E-10	-0.0280	0.0311	0.3668	8.58E-05	38.29
21	rs149449	A	G	0.4892	0.0073	0.0012	2.40E-09	0.0158	0.0299	0.5973	7.99E-05	35.66
22	rs1620977	G	A	0.7305	-0.0132	0.0014	1.10E-21	0.0118	0.0338	0.7278	2.05E-04	91.45
23	rs17049185	T	G	0.2679	0.0080	0.0014	7.30E-09	-0.0004	0.0343	0.9896	7.49E-05	33.44
24	rs1866823	A	G	0.5445	0.0074	0.0012	2.10E-09	-0.0343	0.0307	0.2650	8.04E-05	35.88
25	rs2093654	G	A	0.3881	0.0071	0.0013	1.50E-08	0.0323	0.0310	0.2975	7.18E-05	32.08
26	rs2143081	A	G	0.5398	0.0083	0.0012	1.30E-11	-0.0274	0.0301	0.3635	1.03E-04	45.84
27	rs2790688	T	C	0.1541	0.0114	0.0017	1.50E-11	0.0390	0.0415	0.3470	1.02E-04	45.54
28	rs28479795	T	C	0.2215	0.0112	0.0015	2.50E-14	-0.0736	0.0356	0.0390	1.30E-04	58.11
29	rs329274	G	A	0.4856	0.0068	0.0012	2.80E-08	0.0289	0.0300	0.3356	6.90E-05	30.82
30	rs34162196	T	C	0.1008	-0.0181	0.0020	4.00E-19	0.0187	0.0496	0.7064	1.79E-04	79.89
31	rs4302893	A	G	0.3342	0.0074	0.0013	1.30E-08	-0.0652	0.0320	0.0418	7.24E-05	32.32
32	rs4953150	T	C	0.3441	-0.0084	0.0013	6.60E-11	-0.0013	0.0318	0.9671	9.55E-05	42.62
33	rs559734	C	G	0.7118	0.0078	0.0014	1.10E-08	0.0121	0.0336	0.7190	7.30E-05	32.59
34	rs586346	C	T	0.6354	-0.0069	0.0013	4.50E-08	-0.0417	0.0312	0.1811	6.70E-05	29.90
35	rs60452247	A	G	0.3631	0.0080	0.0013	3.40E-10	-0.0603	0.0311	0.0522	8.83E-05	39.42
36	rs62051554	A	G	0.1085	0.0116	0.0020	4.60E-09	-0.0398	0.0488	0.4145	7.69E-05	34.34
37	rs6475724	T	C	0.7273	0.0077	0.0014	1.90E-08	-0.0164	0.0337	0.6272	7.07E-05	31.58
38	rs72974263	T	C	0.3183	0.0074	0.0013	1.80E-08	-0.0593	0.0322	0.0655	7.10E-05	31.68
39	rs73455661	G	A	0.2793	0.0103	0.0014	4.10E-14	-0.0504	0.0333	0.1301	1.28E-04	57.10
40	rs739320	C	T	0.6058	-0.0090	0.0013	1.90E-12	-0.0267	0.0321	0.4054	1.11E-04	49.64
41	rs7554485	C	T	0.6115	-0.0080	0.0013	1.70E-10	-0.0035	0.0308	0.9096	9.14E-05	40.79
42	rs7818437	C	T	0.2359	-0.0080	0.0015	3.00E-08	-0.0120	0.0360	0.7385	6.88E-05	30.70

43	rs78537042		A	C	0.0868	-0.0119	0.0022	4.80E-08	-0.0034	0.0537	0.9499	6.68E-05	29.81
44	rs7869969		G	A	0.3308	0.0076	0.0013	5.70E-09	0.0434	0.0318	0.1717	7.60E-05	33.93
45	rs7982441		C	T	0.7319	-0.0084	0.0014	9.80E-10	-0.0217	0.0337	0.5195	8.37E-05	37.37
46	rs8095324		G	A	0.4040	-0.0069	0.0012	2.70E-08	0.0069	0.0306	0.8213	6.93E-05	30.93
47	rs817223		C	T	0.4813	-0.0073	0.0012	2.80E-09	0.0010	0.0300	0.9736	7.91E-05	35.30
48	rs862227		G	A	0.4579	-0.0101	0.0012	1.10E-16	-0.0162	0.0299	0.5881	1.54E-04	68.70
49	rs9517948		T	C	0.4514	0.0070	0.0012	1.70E-08	-0.0200	0.0302	0.5085	7.12E-05	31.81
50	rs9919429		G	A	0.4860	-0.0067	0.0012	3.80E-08	0.0121	0.0300	0.6861	6.77E-05	30.23
51	rs994270		G	C	0.2351	0.0133	0.0014	4.20E-20	0.0136	0.0354	0.7013	1.89E-04	84.32
	Vegetable	435435											
1	rs1052352		T	C	0.5236	0.0082	0.0014	1.00E-08	0.0221	0.0300	0.4606	7.53E-05	32.80
2	rs10819082		A	G	0.6673	-0.0092	0.0015	1.40E-09	-0.0044	0.0320	0.8907	8.42E-05	36.67
3	rs12908495		A	C	0.2425	-0.0094	0.0017	2.00E-08	0.0023	0.0351	0.9470	7.24E-05	31.51
4	rs17460017		T	A	0.1901	0.0112	0.0018	7.20E-10	-0.0579	0.0381	0.1280	8.72E-05	37.97
5	rs1890012		G	T	0.1947	-0.0104	0.0018	8.10E-09	0.0268	0.0384	0.4844	7.64E-05	33.25
6	rs3095337		C	G	0.2039	-0.0126	0.0018	9.00E-13	0.0332	0.0372	0.3715	1.17E-04	51.06
7	rs34186148		C	G	0.3701	-0.0081	0.0015	4.80E-08	-0.0012	0.0310	0.9696	6.84E-05	29.80
8	rs4083969		G	C	0.0572	0.0171	0.0031	3.80E-08	-0.0185	0.0669	0.7823	6.95E-05	30.25
9	rs4291983		A	C	0.5176	-0.0084	0.0014	3.70E-09	0.0007	0.0300	0.9802	7.98E-05	34.76
10	rs57221424		G	C	0.3217	0.0089	0.0015	5.50E-09	0.0452	0.0324	0.1626	7.81E-05	34.00
11	rs62461186		C	A	0.1798	-0.0113	0.0019	1.00E-09	-0.0250	0.0392	0.5233	8.57E-05	37.30
12	rs6482190		G	A	0.7194	0.0113	0.0016	1.40E-12	-0.0155	0.0337	0.6460	1.15E-04	50.24
13	rs75248709		T	C	0.0459	-0.0197	0.0035	2.20E-08	-0.0763	0.0787	0.3321	7.19E-05	31.29
14	rs7619139		A	T	0.5892	0.0125	0.0015	8.00E-18	0.0316	0.0306	0.3017	1.70E-04	73.95

15	rs7821179		C	G	0.8466	-0.0108	0.0020	4.40E-08	0.0337	0.0417	0.4195	6.88E-05	29.96
16	rs790561		G	A	0.7041	0.0125	0.0016	1.40E-15	-0.0470	0.0329	0.1523	1.46E-04	63.78
17	rs8130508		A	G	0.2897	0.0087	0.0016	3.00E-08	0.0208	0.0335	0.5355	7.05E-05	30.68
	Green tea	64949											
1	rs113322644		A	C	0.0117	3.2096	0.5551	7.40E-09	-0.0097	0.1410	0.9449	5.15E-04	33.44
2	rs115952340		A	G	0.0133	3.0097	0.5503	4.50E-08	-0.1325	0.1492	0.3745	4.60E-04	29.92
3	rs117077082		G	A	0.0318	1.9206	0.3422	2.00E-08	-0.0185	0.0849	0.8274	4.85E-04	31.50
4	rs117251267		C	T	0.0116	3.5501	0.6178	9.10E-09	0.1221	0.1757	0.4870	5.08E-04	33.03
5	rs11976995		G	T	0.0304	2.0194	0.3487	7.00E-09	0.0141	0.0875	0.8723	5.16E-04	33.54
6	rs12144868		C	T	0.0151	3.0727	0.5275	5.70E-09	0.2336	0.1636	0.1533	5.22E-04	33.94
7	rs12958992		G	A	0.0269	2.1609	0.3789	1.20E-08	0.0593	0.0948	0.5316	5.01E-04	32.52
8	rs142811251		C	G	0.0103	3.4598	0.6178	2.10E-08	0.1324	0.1581	0.4022	4.83E-04	31.36
9	rs189140232		A	G	0.0100	3.5608	0.6257	1.30E-08	0.0813	0.1680	0.6285	4.98E-04	32.39
10	rs62059726		A	G	0.0152	2.8216	0.5118	3.50E-08	-0.1783	0.1306	0.1719	4.68E-04	30.39
11	rs644205		A	G	0.1965	0.8489	0.1508	1.80E-08	0.0210	0.0379	0.5789	4.88E-04	31.71
12	rs78547201		G	C	0.0116	3.2750	0.5777	1.40E-08	-0.0241	0.1547	0.8761	4.95E-04	32.13
13	rs79638269		T	C	0.0163	2.6764	0.4729	1.50E-08	0.0230	0.1147	0.8414	4.93E-04	32.03
	Coffee	428860											
1	rs1057868		T	C	0.2850	0.0200	0.0018	5.40E-29	0.0090	0.0330	0.7844	2.91E-04	124.90
2	rs117968677		A	G	0.0242	-0.0310	0.0055	1.90E-08	0.0242	0.1068	0.8207	7.38E-05	31.65
3	rs12514566		A	G	0.3371	-0.0114	0.0017	2.40E-11	0.0197	0.0316	0.5340	1.04E-04	44.65
4	rs12989746		T	G	0.2499	0.0104	0.0019	2.80E-08	-0.0106	0.0346	0.7603	7.19E-05	30.82
5	rs13054099		C	T	0.2610	-0.0108	0.0018	4.30E-09	-0.0017	0.0341	0.9599	8.03E-05	34.46
6	rs13163336		A	C	0.1576	0.0149	0.0022	1.30E-11	-0.0785	0.0412	0.0571	1.07E-04	45.74

7	rs1338549	G	T	0.5339	-0.0095	0.0016	5.60E-09	-0.0051	0.0301	0.8644	7.92E-05	33.96
8	rs13387939	A	C	0.8284	0.0166	0.0021	9.80E-15	-0.0139	0.0397	0.7269	1.40E-04	59.93
9	rs1421085	C	T	0.4036	0.0185	0.0016	1.70E-29	0.0037	0.0305	0.9040	2.96E-04	127.16
10	rs1527961	C	T	0.1349	-0.0133	0.0024	1.70E-08	0.1011	0.0442	0.0221	7.42E-05	31.81
11	rs17842490	G	A	0.0142	-0.0452	0.0068	3.30E-11	-0.0810	0.1267	0.5227	1.03E-04	44.01
12	rs1942965	C	T	0.5046	-0.0089	0.0016	3.80E-08	-0.0005	0.0301	0.9878	7.05E-05	30.24
13	rs2189234	G	T	0.6178	0.0100	0.0017	1.80E-09	0.0051	0.0308	0.8682	8.43E-05	36.17
14	rs2465037	A	C	0.3430	-0.0106	0.0017	4.80E-10	0.0090	0.0318	0.7783	9.04E-05	38.77
15	rs2472297	T	C	0.2629	0.0465	0.0018	1.10E-142	0.0019	0.0339	0.9553	1.51E-03	646.73
16	rs2597805	T	C	0.6825	0.0099	0.0018	2.00E-08	0.0244	0.0330	0.4610	7.34E-05	31.49
17	rs34060476	G	A	0.1339	0.0184	0.0024	7.50E-15	-0.0247	0.0442	0.5759	1.41E-04	60.45
18	rs4410790	C	T	0.6321	0.0391	0.0017	1.20E-120	-0.0010	0.0311	0.9750	1.27E-03	545.51
19	rs442355	C	G	0.2544	-0.0111	0.0019	1.90E-09	-0.0029	0.0344	0.9322	8.42E-05	36.10
20	rs4615895	A	G	0.7409	0.0122	0.0018	4.20E-11	0.0178	0.0344	0.6045	1.01E-04	43.52
21	rs476828	C	T	0.2374	0.0173	0.0019	5.60E-20	-0.0319	0.0352	0.3636	1.95E-04	83.75
22	rs516636	A	C	0.2089	0.0117	0.0020	4.00E-09	0.0085	0.0367	0.8159	8.07E-05	34.63
23	rs56113850	C	T	0.5781	0.0127	0.0016	8.90E-15	-0.0141	0.0303	0.6405	1.40E-04	60.13
24	rs57918684	A	G	0.1547	0.0129	0.0022	8.60E-09	0.0662	0.0417	0.1127	7.73E-05	33.14
25	rs6062682	T	C	0.4645	0.0104	0.0016	2.50E-10	-0.0051	0.0308	0.8681	9.33E-05	40.02
26	rs6063085	C	A	0.3735	0.0104	0.0017	4.50E-10	-0.0123	0.0309	0.6911	9.07E-05	38.90
27	rs61928609	C	A	0.8353	-0.0147	0.0022	1.30E-11	-0.0645	0.0403	0.1095	1.07E-04	45.85
28	rs62064918	T	C	0.2445	-0.0103	0.0019	4.10E-08	0.0653	0.0348	0.0607	7.02E-05	30.10

29	rs630194		C	T	0.3434	-0.0114	0.0017	2.30E-11	-0.0164	0.0316	0.6029	1.04E-04	44.68
30	rs6469262		C	T	0.5650	-0.0092	0.0016	1.90E-08	-0.0359	0.0302	0.2354	7.36E-05	31.58
31	rs7224815		T	A	0.4078	-0.0109	0.0016	3.70E-11	-0.0656	0.0305	0.0313	1.02E-04	43.77
32	rs73075167		T	A	0.1292	-0.0161	0.0024	5.00E-11	0.0111	0.0462	0.8105	1.01E-04	43.19
33	rs75347775		A	G	0.2445	0.0105	0.0019	2.70E-08	0.0610	0.0348	0.0801	7.21E-05	30.93
34	rs780093		C	T	0.6158	0.0133	0.0017	1.00E-15	-0.0261	0.0307	0.3956	1.50E-04	64.37
35	rs7811609		T	C	0.3747	0.0091	0.0017	4.00E-08	-0.0125	0.0309	0.6842	7.03E-05	30.14
36	rs78267637		G	C	0.0381	-0.0254	0.0043	3.90E-09	-0.0262	0.0814	0.7476	8.09E-05	34.70
37	rs8056750		T	C	0.3591	0.0105	0.0017	1.30E-09	-0.0137	0.0331	0.6776	8.57E-05	36.78
38	rs9398171		T	C	0.7106	0.0109	0.0018	1.10E-09	-0.0058	0.0331	0.8599	8.68E-05	37.21
	Alcohol	462346											
1	rs10188314		T	C	0.4709	-0.0198	0.0030	7.20E-11	-0.0101	0.0302	0.7384	9.19E-05	42.48
2	rs10792669		G	A	0.5053	0.0174	0.0030	9.90E-09	0.0077	0.0302	0.7997	7.11E-05	32.87
3	rs11039429		T	C	0.4546	-0.0236	0.0030	8.70E-15	-0.0005	0.0300	0.9875	1.30E-04	60.16
4	rs11223617		A	G	0.2062	0.0251	0.0038	2.30E-11	0.0035	0.0371	0.9245	9.66E-05	44.68
5	rs11700855		G	A	0.0935	-0.0298	0.0052	1.20E-08	0.0716	0.0519	0.1679	7.01E-05	32.42
6	rs11750777		A	G	0.2095	-0.0205	0.0037	3.80E-08	-0.0371	0.0368	0.3145	6.54E-05	30.25
7	rs11787216		T	C	0.3691	0.0244	0.0032	2.40E-14	0.0069	0.0322	0.8311	1.26E-04	58.19
8	rs11940694		G	A	0.6042	-0.0437	0.0031	1.00E-44	0.0122	0.0311	0.6950	4.25E-04	196.80
9	rs12153855		C	T	0.1050	0.0294	0.0049	2.40E-09	-0.0264	0.0491	0.5900	7.70E-05	35.60
10	rs1228589		A	G	0.2461	0.0211	0.0035	2.30E-09	0.0749	0.0351	0.0329	7.71E-05	35.67
11	rs1229984		C	T	0.9728	-0.2617	0.0092	1.40E-178	-0.0728	0.0945	0.4412	1.75E-03	811.86
12	rs12312693		C	T	0.4518	-0.0177	0.0031	6.80E-09	0.0813	0.0303	0.0073	7.27E-05	33.60

13	rs13102973	C	T	0.6188	-0.0194	0.0031	4.90E-10	0.0126	0.0309	0.6835	8.37E-05	38.72
14	rs13135092	G	A	0.0835	0.0438	0.0055	1.60E-15	0.0506	0.0546	0.3534	1.37E-04	63.55
15	rs13178443	T	C	0.2763	-0.0187	0.0034	3.80E-08	-0.0147	0.0335	0.6614	6.55E-05	30.27
16	rs13390019	C	T	0.1340	0.0296	0.0045	4.30E-11	0.0086	0.0450	0.8480	9.40E-05	43.46
17	rs1421085	C	T	0.4034	0.0199	0.0031	1.00E-10	0.0037	0.0305	0.9040	9.04E-05	41.78
18	rs1515591	G	T	0.3832	0.0182	0.0031	4.90E-09	0.0209	0.0309	0.4984	7.40E-05	34.22
19	rs1666658	C	T	0.3922	0.0180	0.0031	6.70E-09	0.0213	0.0306	0.4879	7.27E-05	33.62
20	rs17662759	C	T	0.0891	0.0301	0.0055	3.40E-08	0.0146	0.0556	0.7926	6.59E-05	30.46
21	rs17690703	T	C	0.2627	0.0250	0.0034	2.90E-13	0.0082	0.0338	0.8080	1.15E-04	53.26
22	rs186347	T	G	0.4633	0.0179	0.0031	4.00E-09	0.0360	0.0303	0.2354	7.49E-05	34.62
23	rs1893659	A	C	0.4599	-0.0293	0.0031	7.60E-22	-0.0031	0.0303	0.9176	2.00E-04	92.26
24	rs1937522	G	A	0.5281	0.0169	0.0030	2.50E-08	-0.0031	0.0300	0.9183	6.72E-05	31.06
25	rs1991083	T	C	0.6799	-0.0224	0.0033	6.30E-12	-0.0302	0.0323	0.3511	1.02E-04	47.24
26	rs2043677	T	C	0.1456	0.0261	0.0043	1.60E-09	0.0308	0.0434	0.4772	7.88E-05	36.42
27	rs2159935	A	G	0.4904	-0.0186	0.0030	8.30E-10	0.0504	0.0300	0.0925	8.15E-05	37.68
28	rs2160935	T	C	0.6043	-0.0187	0.0031	1.40E-09	-0.0526	0.0306	0.0854	7.93E-05	36.67
29	rs2244598	C	T	0.6051	-0.0184	0.0031	3.80E-09	-0.0954	0.0310	0.0021	7.51E-05	34.72
30	rs2411453	G	T	0.5974	-0.0351	0.0031	7.30E-30	-0.0305	0.0307	0.3195	2.79E-04	128.85
31	rs2535911	T	C	0.3547	-0.0188	0.0032	2.70E-09	-0.0216	0.0313	0.4896	7.65E-05	35.38
32	rs2622167	A	G	0.4287	-0.0191	0.0031	4.60E-10	-0.0071	0.0305	0.8147	8.40E-05	38.84
33	rs262240	T	C	0.4686	-0.0172	0.0030	1.40E-08	0.0453	0.0300	0.1312	6.95E-05	32.15
34	rs2717063	A	C	0.5857	-0.0204	0.0031	4.00E-11	-0.0009	0.0308	0.9770	9.43E-05	43.61
35	rs28622224	T	C	0.2804	-0.0186	0.0034	3.20E-08	0.0663	0.0334	0.0469	6.61E-05	30.56
36	rs28768122	C	T	0.7595	0.0207	0.0036	5.60E-09	-0.0068	0.0352	0.8464	7.34E-05	33.96

37	rs28787109	A	G	0.4042	0.0178	0.0031	7.70E-09	0.0039	0.0306	0.8992	7.21E-05	33.34
38	rs2924321	A	G	0.5396	-0.0195	0.0031	1.60E-10	-0.0428	0.0303	0.1578	8.85E-05	40.93
39	rs2977454	G	C	0.1241	-0.0259	0.0046	1.70E-08	-0.0120	0.0455	0.7922	6.87E-05	31.78
40	rs34440851	T	C	0.1572	-0.0227	0.0042	4.60E-08	0.0643	0.0410	0.1168	6.46E-05	29.87
41	rs34473884	A	G	0.2482	-0.0204	0.0035	6.20E-09	-0.0498	0.0347	0.1506	7.31E-05	33.78
42	rs34631026	T	C	0.4461	-0.0169	0.0030	2.90E-08	-0.0209	0.0301	0.4884	6.66E-05	30.78
43	rs34811474	A	G	0.2307	-0.0202	0.0036	1.90E-08	-0.0463	0.0355	0.1917	6.82E-05	31.55
44	rs35105141	T	C	0.4015	0.0263	0.0031	1.40E-17	0.0085	0.0306	0.7803	1.57E-04	72.79
45	rs362307	T	C	0.0746	0.0433	0.0058	8.40E-14	0.0053	0.0583	0.9275	1.20E-04	55.70
46	rs4241258	T	C	0.1376	0.0251	0.0044	1.30E-08	0.0196	0.0436	0.6539	7.01E-05	32.40
47	rs4242715	A	G	0.6806	-0.0187	0.0032	9.30E-09	-0.0370	0.0321	0.2495	7.13E-05	32.98
48	rs4417025	A	G	0.3612	-0.0188	0.0032	2.70E-09	0.0364	0.0313	0.2449	7.66E-05	35.42
49	rs4503294	T	C	0.5653	0.0181	0.0031	3.40E-09	-0.0201	0.0305	0.5098	7.55E-05	34.93
50	rs461599	C	A	0.4623	-0.0192	0.0030	2.70E-10	-0.0199	0.0301	0.5075	8.62E-05	39.85
51	rs4726481	T	G	0.4006	0.0218	0.0031	2.30E-12	-0.0431	0.0309	0.1630	1.06E-04	49.22
52	rs473098	T	C	0.5577	-0.0217	0.0030	9.10E-13	0.0196	0.0301	0.5149	1.10E-04	51.03
53	rs489062	A	G	0.4375	0.0166	0.0031	4.90E-08	0.0276	0.0301	0.3597	6.43E-05	29.74
54	rs4916723	C	A	0.4206	0.0239	0.0031	1.10E-14	0.0034	0.0310	0.9130	1.29E-04	59.70
55	rs4940926	C	T	0.7350	-0.0191	0.0034	2.80E-08	0.0082	0.0340	0.8090	6.66E-05	30.82
56	rs4968391	T	G	0.6749	-0.0193	0.0032	2.30E-09	-0.0699	0.0319	0.0287	7.71E-05	35.67
57	rs550942	T	C	0.8239	0.0224	0.0040	2.00E-08	0.0087	0.0394	0.8246	6.82E-05	31.54
58	rs56194430	T	C	0.1693	0.0225	0.0041	3.10E-08	0.0153	0.0408	0.7070	6.63E-05	30.65
59	rs58905411	A	G	0.4101	-0.0266	0.0031	5.10E-18	0.0164	0.0305	0.5912	1.62E-04	74.85
60	rs6030200	A	G	0.3142	-0.0195	0.0033	2.40E-09	0.0912	0.0325	0.0050	7.71E-05	35.65

61	rs61873510	T	G	0.3279	0.0204	0.0033	6.90E-10	-0.0440	0.0336	0.1904	8.23E-05	38.04
62	rs62305780	G	C	0.1023	-0.0485	0.0051	9.90E-22	-0.0352	0.0513	0.4928	1.98E-04	91.74
63	rs62339673	A	C	0.6267	0.0183	0.0032	6.60E-09	-0.0329	0.0315	0.2967	7.28E-05	33.64
64	rs62466318	T	C	0.2028	-0.0255	0.0038	1.40E-11	0.0066	0.0375	0.8594	9.87E-05	45.62
65	rs650558	T	C	0.2479	0.0207	0.0035	3.40E-09	0.0361	0.0347	0.2984	7.56E-05	34.94
66	rs6727281	T	C	0.1840	-0.0243	0.0039	5.50E-10	-0.0074	0.0390	0.8496	8.33E-05	38.50
67	rs6943160	C	T	0.2086	0.0206	0.0037	3.10E-08	0.0132	0.0370	0.7214	6.62E-05	30.62
68	rs71651683	T	C	0.0142	-0.0705	0.0128	3.60E-08	-0.0969	0.1267	0.4444	6.56E-05	30.35
69	rs72769229	T	A	0.1549	-0.0231	0.0042	3.40E-08	-0.0893	0.0415	0.0314	6.59E-05	30.46
70	rs72787062	A	G	0.1628	-0.0282	0.0041	6.40E-12	0.0025	0.0407	0.9517	1.02E-04	47.22
71	rs728538	G	T	0.1689	0.0229	0.0041	1.80E-08	0.0028	0.0405	0.9441	6.86E-05	31.70
72	rs7298932	G	A	0.1478	-0.0237	0.0043	3.80E-08	-0.0684	0.0431	0.1122	6.55E-05	30.27
73	rs7302200	A	G	0.3400	-0.0184	0.0032	8.40E-09	0.0104	0.0317	0.7427	7.17E-05	33.18
74	rs73050128	A	C	0.1645	-0.0260	0.0041	2.10E-10	-0.0113	0.0405	0.7810	8.74E-05	40.41
75	rs7330939	T	C	0.7204	-0.0213	0.0034	3.70E-10	0.0557	0.0340	0.1009	8.49E-05	39.25
76	rs74679146	C	T	0.0745	-0.0321	0.0058	2.50E-08	-0.0997	0.0568	0.0791	6.71E-05	31.03
77	rs7514579	C	A	0.2325	0.0197	0.0036	4.60E-08	-0.0347	0.0357	0.3318	6.46E-05	29.88
78	rs76082653	T	C	0.0543	0.0464	0.0067	3.80E-12	-0.0304	0.0662	0.6458	1.04E-04	48.21
79	rs7610856	A	C	0.4291	-0.0239	0.0031	7.70E-15	-0.0294	0.0305	0.3353	1.31E-04	60.42
80	rs780094	C	T	0.6152	-0.0510	0.0031	1.30E-60	-0.0280	0.0307	0.3622	5.83E-04	269.71
81	rs780569	A	T	0.7088	0.0198	0.0034	4.00E-09	-0.0162	0.0337	0.6319	7.49E-05	34.64
82	rs80292319	C	T	0.0577	-0.0394	0.0065	1.40E-09	0.0588	0.0639	0.3572	7.95E-05	36.74
83	rs8043563	C	G	0.7372	0.0234	0.0035	1.70E-11	0.0100	0.0346	0.7717	9.80E-05	45.31
84	rs838145	A	G	0.5430	0.0220	0.0031	6.70E-13	-0.0048	0.0303	0.8733	1.12E-04	51.63

85	rs8614		A	C	0.1825	0.0248	0.0039	2.70E-10	0.0517	0.0389	0.1843	8.62E-05	39.85
86	rs9349379		G	A	0.4055	-0.0193	0.0031	3.50E-10	0.0182	0.0304	0.5501	8.52E-05	39.40
87	rs9372625		A	G	0.3817	-0.0256	0.0031	2.90E-16	-0.0031	0.0310	0.9200	1.45E-04	66.90
88	rs9403297		A	G	0.3730	0.0188	0.0031	1.80E-09	-0.0599	0.0310	0.0532	7.82E-05	36.16
89	rs9648478		A	G	0.5102	0.0169	0.0030	2.60E-08	0.0090	0.0300	0.7637	6.70E-05	30.99
90	rs9814516		T	G	0.2374	-0.0251	0.0036	1.60E-12	0.0228	0.0352	0.5163	1.08E-04	49.87
91	rs9829192		T	G	0.4351	0.0169	0.0031	2.80E-08	-0.0273	0.0302	0.3665	6.66E-05	30.81
92	rs9906502		A	G	0.1770	0.0238	0.0040	1.90E-09	0.0418	0.0393	0.2878	7.80E-05	36.05
93	rs9912298		C	A	0.2396	0.0206	0.0036	9.70E-09	0.0577	0.0359	0.1085	7.11E-05	32.89
	Principal component-derived dietary pattern 1	449210											
1	rs1013221		C	A	0.5957	0.0264	0.0048	3.20E-08	-0.0280	0.0305	0.3589	6.80E-05	30.55
2	rs10172475		G	A	0.5729	0.0258	0.0047	3.60E-08	0.0508	0.0302	0.0926	6.68E-05	30.02
3	rs10189857		A	G	0.5677	0.0304	0.0047	1.10E-10	-0.0740	0.0302	0.0143	9.22E-05	41.41
4	rs10206196		T	C	0.5420	-0.0299	0.0047	2.10E-10	0.0481	0.0301	0.1102	9.04E-05	40.63
5	rs10249294		G	A	0.6267	-0.0276	0.0048	1.40E-08	-0.0254	0.0309	0.4118	7.27E-05	32.65
6	rs10253861		G	A	0.4478	-0.0261	0.0047	3.50E-08	-0.0021	0.0302	0.9434	6.85E-05	30.75
7	rs10745324		A	G	0.3521	0.0285	0.0049	7.80E-09	0.0211	0.0313	0.5014	7.59E-05	34.10
8	rs10765776		C	A	0.6091	-0.0358	0.0048	6.20E-14	0.0227	0.0309	0.4621	1.23E-04	55.28
9	rs111915841		G	C	0.6774	-0.0349	0.0050	2.70E-12	0.0551	0.0324	0.0885	1.07E-04	48.29
10	rs112060520		G	T	0.7021	-0.0329	0.0051	3.00E-10	-0.0413	0.0329	0.2096	9.20E-05	41.33
11	rs1136666		C	G	0.7234	0.0315	0.0053	3.80E-09	-0.0112	0.0340	0.7412	7.93E-05	35.61
12	rs114340522		G	C	0.9510	-0.0624	0.0109	8.80E-09	-0.0684	0.0706	0.3327	7.27E-05	32.66

13	rs114672787	T	G	0.9452	-0.0562	0.0103	3.80E-08	0.0452	0.0660	0.4927	6.66E-05	29.93
14	rs1147198	G	T	0.2309	-0.0329	0.0055	3.10E-09	0.0368	0.0354	0.2986	7.86E-05	35.31
15	rs11693885	G	A	0.5527	0.0293	0.0047	3.40E-10	-0.0181	0.0304	0.5526	8.54E-05	38.35
16	rs1176799	C	A	0.6272	0.0268	0.0048	3.20E-08	-0.0170	0.0309	0.5820	6.82E-05	30.65
17	rs11772223	A	G	0.8064	-0.0328	0.0059	4.40E-08	-0.0105	0.0379	0.7826	6.84E-05	30.75
18	rs11790060	T	C	0.6685	-0.0295	0.0050	3.10E-09	-0.0453	0.0318	0.1538	7.84E-05	35.22
19	rs1183668	C	G	0.6301	-0.0285	0.0049	5.50E-09	0.0409	0.0313	0.1923	7.62E-05	34.23
20	rs11852327	C	T	0.7396	-0.0478	0.0053	2.50E-19	-0.0027	0.0343	0.9379	1.78E-04	80.18
21	rs12029204	G	A	0.2991	0.0291	0.0051	1.20E-08	0.0026	0.0327	0.9373	7.25E-05	32.56
22	rs12485729	G	A	0.6507	0.0271	0.0049	2.50E-08	0.0242	0.0316	0.4443	6.75E-05	30.32
23	rs12546366	T	C	0.4519	0.0377	0.0047	1.50E-15	-0.0514	0.0302	0.0889	1.42E-04	63.83
24	rs12793712	C	T	0.7049	0.0331	0.0052	1.00E-10	-0.0062	0.0331	0.8518	9.19E-05	41.27
25	rs12896620	C	T	0.2786	-0.0302	0.0052	6.10E-09	-0.0054	0.0335	0.8718	7.41E-05	33.30
26	rs12953563	C	T	0.5802	-0.0318	0.0048	2.30E-11	0.0157	0.0304	0.6064	9.95E-05	44.69
27	rs12968428	A	G	0.6181	-0.0287	0.0048	2.30E-09	0.0030	0.0309	0.9231	7.90E-05	35.49
28	rs12969294	A	G	0.3390	-0.0337	0.0050	5.30E-12	0.0008	0.0317	0.9797	1.03E-04	46.21
29	rs13064780	T	G	0.3760	-0.0442	0.0054	4.10E-16	-0.0218	0.0309	0.4819	1.49E-04	67.11
30	rs13392900	A	G	0.9316	-0.0530	0.0093	1.40E-08	-0.0166	0.0597	0.7813	7.21E-05	32.38
31	rs138637028	C	T	0.9825	0.1005	0.0180	2.60E-08	-0.0170	0.1167	0.8845	6.95E-05	31.21
32	rs1394257	G	T	0.7231	-0.0315	0.0052	2.70E-09	0.0491	0.0336	0.1441	8.09E-05	36.34
33	rs1402954	C	T	0.9093	-0.0441	0.0081	3.80E-08	-0.0724	0.0521	0.1649	6.54E-05	29.36
34	rs1421085	T	C	0.5969	-0.0659	0.0048	2.30E-43	-0.0037	0.0305	0.9040	4.25E-04	191.03
35	rs16854051	C	T	0.7926	0.0382	0.0058	5.90E-11	-0.0025	0.0376	0.9479	9.61E-05	43.16
36	rs17782313	T	C	0.7670	-0.0593	0.0055	8.00E-27	0.0333	0.0353	0.3457	2.56E-04	115.06

37	rs1857952	A	C	0.4548	-0.0316	0.0047	2.70E-11	-0.0243	0.0306	0.4259	9.88E-05	44.40
38	rs192070971	G	A	0.5208	-0.0304	0.0047	4.60E-11	0.0689	0.0301	0.0222	9.32E-05	41.86
39	rs202637	A	G	0.1830	0.0354	0.0061	5.20E-09	-0.0459	0.0390	0.2389	7.54E-05	33.89
40	rs2417261	T	G	0.1070	-0.0422	0.0076	2.30E-08	-0.0097	0.0488	0.8415	6.85E-05	30.77
41	rs2422853	T	C	0.3865	-0.0265	0.0049	3.80E-08	0.0145	0.0313	0.6439	6.63E-05	29.81
42	rs2429056	A	C	0.6258	-0.0291	0.0048	1.40E-09	0.0119	0.0309	0.7006	8.09E-05	36.32
43	rs2742690	C	A	0.7911	0.0329	0.0058	1.20E-08	-0.0283	0.0376	0.4510	7.15E-05	32.10
44	rs2817399	A	G	0.5367	0.0293	0.0047	5.00E-10	-0.0336	0.0301	0.2649	8.67E-05	38.95
45	rs28703977	T	C	0.3453	0.0353	0.0049	8.10E-13	-0.0001	0.0316	0.9974	1.14E-04	51.14
46	rs28869247	A	G	0.6357	0.0301	0.0049	1.40E-09	0.0199	0.0314	0.5274	8.41E-05	37.80
47	rs3101336	T	C	0.3977	-0.0599	0.0048	1.80E-36	0.0005	0.0306	0.9880	3.51E-04	157.86
48	rs35203607	G	T	0.8285	-0.0356	0.0062	1.30E-08	-0.0075	0.0400	0.8503	7.28E-05	32.68
49	rs35287743	G	T	0.8837	0.0581	0.0074	6.60E-15	-0.0516	0.0474	0.2766	1.39E-04	62.34
50	rs36086644	A	G	0.4889	0.0285	0.0047	1.30E-09	-0.0129	0.0300	0.6677	8.24E-05	37.02
51	rs3747631	G	C	0.7906	-0.0388	0.0057	1.20E-11	0.0135	0.0367	0.7139	1.02E-04	45.92
52	rs384317	G	A	0.5547	0.0293	0.0047	4.00E-10	-0.0375	0.0301	0.2130	8.64E-05	38.82
53	rs3845	T	C	0.2466	-0.0413	0.0054	5.30E-14	-0.0251	0.0350	0.4739	1.28E-04	57.60
54	rs4024198	C	G	0.2927	-0.0376	0.0051	2.70E-13	0.0454	0.0330	0.1682	1.19E-04	53.36
55	rs429358	T	C	0.8453	-0.0766	0.0065	1.10E-32	0.0091	0.0414	0.8254	3.11E-04	139.96
56	rs4489409	C	T	0.6242	0.0367	0.0048	5.40E-14	0.0111	0.0309	0.7192	1.29E-04	57.77
57	rs4625545	G	A	0.6623	-0.0282	0.0049	1.20E-08	0.0119	0.0317	0.7071	7.20E-05	32.35
58	rs4852285	A	G	0.6690	-0.0314	0.0050	4.00E-10	0.0150	0.0319	0.6377	8.87E-05	39.86
59	rs4853701	T	C	0.3185	-0.0329	0.0050	5.50E-11	-0.0184	0.0321	0.5661	9.55E-05	42.90
60	rs4953150	C	T	0.6557	0.0346	0.0049	1.40E-12	0.0013	0.0318	0.9671	1.09E-04	48.95

61	rs4963390	A	G	0.8612	-0.0380	0.0068	3.10E-08	0.0468	0.0436	0.2835	6.98E-05	31.35
62	rs56210058	G	A	0.8069	0.0327	0.0060	4.40E-08	0.0020	0.0383	0.9592	6.70E-05	30.12
63	rs5753630	A	G	0.5592	-0.0316	0.0047	2.50E-11	-0.0420	0.0303	0.1661	9.94E-05	44.65
64	rs578096	T	C	0.1603	0.0417	0.0064	6.60E-11	0.0140	0.0408	0.7307	9.57E-05	42.97
65	rs57912727	A	C	0.8641	0.0367	0.0068	4.20E-08	0.0321	0.0440	0.4649	6.41E-05	28.78
66	rs6024408	C	T	0.7011	0.0323	0.0051	2.40E-10	0.0130	0.0327	0.6902	8.88E-05	39.88
67	rs6088765	T	G	0.5818	-0.0262	0.0048	4.10E-08	0.0190	0.0303	0.5300	6.78E-05	30.45
68	rs61519036	C	T	0.6608	-0.0269	0.0050	4.10E-08	0.0467	0.0322	0.1474	6.50E-05	29.21
69	rs62473709	A	G	0.1694	0.0370	0.0062	3.80E-09	-0.0112	0.0399	0.7795	7.83E-05	35.19
70	rs6751993	A	G	0.1720	-0.0551	0.0063	1.30E-18	0.0085	0.0406	0.8348	1.72E-04	77.13
71	rs6767669	G	A	0.7293	0.0313	0.0054	4.70E-09	-0.0697	0.0357	0.0506	7.49E-05	33.64
72	rs6808936	A	G	0.5552	-0.0278	0.0047	5.60E-09	0.0242	0.0301	0.4226	7.80E-05	35.02
73	rs6843563	A	G	0.3421	-0.0278	0.0049	1.00E-08	0.0667	0.0315	0.0346	7.07E-05	31.74
74	rs6851174	G	T	0.6398	0.0270	0.0049	1.70E-08	0.0385	0.0312	0.2170	6.84E-05	30.73
75	rs6882046	A	G	0.7336	-0.0378	0.0053	2.60E-12	0.0328	0.0346	0.3431	1.12E-04	50.14
76	rs6969458	G	A	0.5323	0.0307	0.0047	5.90E-11	0.0244	0.0305	0.4237	9.39E-05	42.20
77	rs7132908	G	A	0.6155	-0.0279	0.0048	6.10E-09	0.0630	0.0307	0.0404	7.50E-05	33.68
78	rs7213721	G	T	0.5380	0.0266	0.0047	1.10E-08	-0.0731	0.0301	0.0151	7.15E-05	32.11
79	rs7253058	T	G	0.8754	-0.0387	0.0071	4.50E-08	0.0267	0.0461	0.5632	6.57E-05	29.49
80	rs72673546	C	T	0.9018	0.0515	0.0079	6.80E-11	-0.0505	0.0503	0.3150	9.55E-05	42.88
81	rs72720396	A	G	0.7700	-0.0421	0.0055	2.80E-14	0.0141	0.0355	0.6926	1.28E-04	57.70
82	rs72900104	G	A	0.6808	-0.0308	0.0051	1.30E-09	0.0544	0.0329	0.0988	8.11E-05	36.45
83	rs7469124	G	A	0.3880	0.0303	0.0048	4.10E-10	-0.0133	0.0309	0.6680	8.81E-05	39.58
84	rs7497801	G	A	0.6102	0.0264	0.0048	4.00E-08	0.0081	0.0307	0.7912	6.74E-05	30.28

85	rs75440569		G	A	0.9253	0.0504	0.0090	2.30E-08	0.0379	0.0578	0.5127	7.03E-05	31.60
86	rs7609489		T	A	0.6154	0.0330	0.0049	1.50E-11	-0.0095	0.0316	0.7633	1.03E-04	46.19
87	rs76204832		C	A	0.6779	-0.0277	0.0050	2.90E-08	0.0291	0.0322	0.3661	6.81E-05	30.58
88	rs76729647		G	A	0.9343	-0.0524	0.0094	2.50E-08	-0.0134	0.0604	0.8240	6.87E-05	30.87
89	rs77165542		C	T	0.9645	0.0902	0.0128	1.90E-12	-0.0551	0.0826	0.5047	1.11E-04	49.84
90	rs79638430		G	A	0.7364	-0.0298	0.0054	3.00E-08	0.0179	0.0344	0.6031	6.88E-05	30.90
91	rs806789		C	A	0.5298	-0.0383	0.0047	2.00E-16	-0.0482	0.0299	0.1077	1.49E-04	66.78
92	rs898751		C	T	0.5074	0.0322	0.0047	5.80E-12	-0.0108	0.0300	0.7187	1.05E-04	47.33
93	rs909334		C	A	0.7517	-0.0310	0.0054	1.50E-08	0.0517	0.0346	0.1354	7.28E-05	32.69
94	rs9320823		T	C	0.3976	0.0365	0.0048	1.70E-14	-0.0250	0.0306	0.4144	1.30E-04	58.26
95	rs9422753		T	C	0.8100	-0.0427	0.0060	1.30E-12	0.0051	0.0382	0.8937	1.14E-04	51.03
96	rs9436758		A	G	0.9160	-0.0509	0.0084	1.50E-09	-0.0236	0.0540	0.6626	8.11E-05	36.42
97	rs956362		A	G	0.7883	-0.0341	0.0058	3.40E-09	0.0198	0.0371	0.5930	7.71E-05	34.64
98	rs9585326		A	G	0.4664	-0.0269	0.0047	9.60E-09	0.0264	0.0300	0.3788	7.32E-05	32.86
99	rs958661		T	C	0.6390	0.0316	0.0049	6.70E-11	-0.0573	0.0313	0.0673	9.31E-05	41.84
100	rs9825480		G	A	0.5210	-0.0279	0.0047	1.80E-09	0.0240	0.0306	0.4330	7.78E-05	34.97
	Highly palatable diet pattern	158666											
1	rs10128495		T	G	0.3346	0.0222	0.0038	3.75E-09	0.0683	0.0319	0.0325	2.19E-04	34.75
2	rs12195577		C	T	0.4596	-0.0207	0.0036	6.22E-09	-0.0072	0.0309	0.8153	2.13E-04	33.76
3	rs2043539		A	G	0.4178	-0.0202	0.0036	2.01E-08	-0.0111	0.0303	0.7142	1.98E-04	31.48
4	rs2470578		A	G	0.4899	0.0218	0.0035	7.89E-10	0.0207	0.0301	0.4913	2.38E-04	37.79
5	rs2945319		C	T	0.4731	-0.0255	0.0036	7.09E-13	-0.0263	0.0307	0.3919	3.25E-04	51.52
6	rs35626515		A	C	0.4032	0.0235	0.0036	8.46E-11	0.0284	0.0306	0.3534	2.66E-04	42.15

7	rs4649981		A	G	0.4664	-0.0232	0.0036	6.47E-11	0.0389	0.0300	0.1957	2.69E-04	42.67
8	rs55873205		G	C	0.2777	-0.0244	0.0040	7.45E-10	0.0047	0.0337	0.8899	2.39E-04	37.90
9	rs6673455		C	T	0.3976	-0.0219	0.0036	1.58E-09	-0.0579	0.0306	0.0589	2.30E-04	36.43
10	rs6985751		G	A	0.4441	0.0236	0.0036	3.98E-11	0.0890	0.0302	0.0032	2.75E-04	43.62
11	rs73288458		G	C	0.3103	-0.0212	0.0038	3.49E-08	0.0199	0.0329	0.5447	1.92E-04	30.41
12	rs7587672		C	G	0.2606	-0.0228	0.0040	1.63E-08	-0.0373	0.0343	0.2771	2.01E-04	31.90
13	rs76395182		G	T	0.3529	-0.0240	0.0037	1.05E-10	0.0096	0.0324	0.7677	2.63E-04	41.72
14	rs7712317		C	T	0.1665	0.0271	0.0048	1.22E-08	0.0536	0.0405	0.1860	2.05E-04	32.46
15	rs77735344		T	A	0.0759	-0.0366	0.0067	4.51E-08	-0.0661	0.0594	0.2654	1.89E-04	29.92
16	rs814573		T	A	0.1806	-0.0454	0.0046	7.60E-23	-0.0239	0.0400	0.5498	6.10E-04	96.82
17	rs9397584		T	C	0.2834	0.0226	0.0039	9.31E-09	0.0478	0.0331	0.1491	2.08E-04	32.98
	Low caloric diet pattern	154998											
1	rs10898126		T	G	0.4069	-0.0760	0.0134	1.51E-08	0.0865	0.0305	0.0046	2.07E-04	32.04
2	rs11879227		G	A	0.1835	0.1034	0.0170	1.30E-09	-0.0151	0.0387	0.6962	2.37E-04	36.81
3	rs224111		A	G	0.3913	-0.0745	0.0135	3.58E-08	0.0000	0.0309	0.9998	1.96E-04	30.37
4	rs4364183		A	G	0.2857	-0.0979	0.0146	2.04E-11	0.0567	0.0332	0.0875	2.90E-04	44.93
5	rs4486206		G	A	0.2332	0.0859	0.0156	3.66E-08	0.0159	0.0358	0.6565	1.96E-04	30.32
6	rs933738		G	A	0.1728	0.1025	0.0174	4.24E-09	-0.0690	0.0398	0.0829	2.23E-04	34.51
7	rs9537116		A	G	0.2656	0.1171	0.0149	4.43E-15	0.0346	0.0347	0.3187	3.97E-04	61.50
	Acquired diet pattern	156639											
1	rs10119226		C	G	0.4155	-0.0577	0.0094	9.85E-10	0.0141	0.0305	0.6437	2.38E-04	37.35
2	rs10124367		T	C	0.3679	-0.0540	0.0096	2.18E-08	0.0029	0.0310	0.9254	2.00E-04	31.33

3	rs10160758	C	T	0.2680	-0.0618	0.0105	3.95E-09	0.0795	0.0343	0.0204	2.21E-04	34.65
4	rs10195287	T	C	0.4235	-0.0571	0.0094	1.32E-09	0.0301	0.0303	0.3209	2.35E-04	36.78
5	rs10839921	T	C	0.3571	0.0546	0.0097	1.89E-08	-0.0304	0.0323	0.3459	2.02E-04	31.60
6	rs11012794	G	A	0.2932	0.0595	0.0102	5.70E-09	-0.0464	0.0331	0.1610	2.17E-04	33.94
7	rs1123321	G	C	0.2579	0.0632	0.0106	2.75E-09	-0.0311	0.0346	0.3694	2.26E-04	35.35
8	rs11638106	T	C	0.4434	0.0522	0.0094	2.45E-08	-0.0246	0.0301	0.4147	1.99E-04	31.10
9	rs11790555	C	T	0.1985	0.0710	0.0117	1.15E-09	-0.0075	0.0377	0.8432	2.37E-04	37.06
10	rs12455474	G	T	0.4015	0.0554	0.0095	5.10E-09	0.0042	0.0306	0.8913	2.18E-04	34.15
11	rs13262595	G	A	0.4363	0.0559	0.0094	2.45E-09	0.0815	0.0301	0.0069	2.27E-04	35.58
12	rs138799955	G	A	0.2748	0.0648	0.0104	5.08E-10	-0.0139	0.0353	0.6934	2.47E-04	38.65
13	rs1391438	C	T	0.3137	-0.0562	0.0100	2.07E-08	-0.0175	0.0323	0.5875	2.01E-04	31.43
14	rs1937522	G	A	0.4742	-0.0530	0.0093	1.27E-08	-0.0031	0.0300	0.9183	2.07E-04	32.38
15	rs2236295	T	G	0.3992	0.0734	0.0095	1.06E-14	-0.0211	0.0307	0.4915	3.82E-04	59.79
16	rs3105049	T	C	0.2715	-0.1067	0.0105	1.90E-24	-0.0282	0.0338	0.4032	6.64E-04	104.12
17	rs34388845	G	A	0.2125	-0.0752	0.0114	3.67E-11	-0.0186	0.0365	0.6099	2.79E-04	43.78
18	rs4684095	C	T	0.2845	0.0568	0.0103	3.59E-08	0.0158	0.0331	0.6334	1.94E-04	30.36
19	rs4858697	G	A	0.4180	0.0640	0.0094	1.11E-11	0.0416	0.0306	0.1739	2.94E-04	46.13
20	rs504675	T	C	0.3508	0.0686	0.0097	1.92E-12	0.0376	0.0314	0.2317	3.16E-04	49.56
21	rs511450	A	G	0.2923	-0.0603	0.0102	3.61E-09	-0.0197	0.0329	0.5489	2.22E-04	34.82
22	rs55804009	A	C	0.1401	0.0835	0.0134	4.57E-10	0.0103	0.0435	0.8126	2.48E-04	38.85
23	rs55872725	T	C	0.4046	0.0719	0.0095	3.25E-14	0.0037	0.0305	0.9024	3.67E-04	57.58
24	rs6791295	G	A	0.3648	0.0559	0.0097	7.08E-09	0.0038	0.0312	0.9030	2.14E-04	33.51
25	rs6841352	T	G	0.4615	-0.0546	0.0093	4.70E-09	0.0235	0.0301	0.4340	2.19E-04	34.31
26	rs7610856	A	C	0.4323	0.0622	0.0094	3.56E-11	-0.0294	0.0305	0.3353	2.80E-04	43.84

27	rs7652683	C	T	0.3500	-0.0911	0.0097	8.92E-21	-0.0398	0.0313	0.2039	5.58E-04	87.39
28	rs7655455	T	C	0.3378	0.0673	0.0098	7.82E-12	-0.0483	0.0317	0.1279	2.99E-04	46.81
29	rs786272	A	G	0.3400	-0.0569	0.0098	6.83E-09	0.0152	0.0318	0.6335	2.14E-04	33.58
30	rs7991820	C	A	0.2556	0.0597	0.0107	2.13E-08	0.0456	0.0343	0.1841	2.00E-04	31.37
31	rs80028338	C	A	0.2051	-0.0991	0.0115	7.39E-18	-0.0009	0.0403	0.9816	4.73E-04	74.11
32	rs810368	A	G	0.1619	-0.0698	0.0126	3.23E-08	0.0157	0.0417	0.7068	1.95E-04	30.57
33	rs814573	T	A	0.1806	-0.0829	0.0121	6.99E-12	-0.0239	0.0400	0.5498	3.00E-04	47.03
34	rs922234	G	C	0.3326	0.0722	0.0099	2.57E-13	-0.0267	0.0319	0.4013	3.42E-04	53.51
35	rs9817162	T	C	0.4333	0.0583	0.0094	5.18E-10	-0.0162	0.0302	0.5903	2.46E-04	38.61

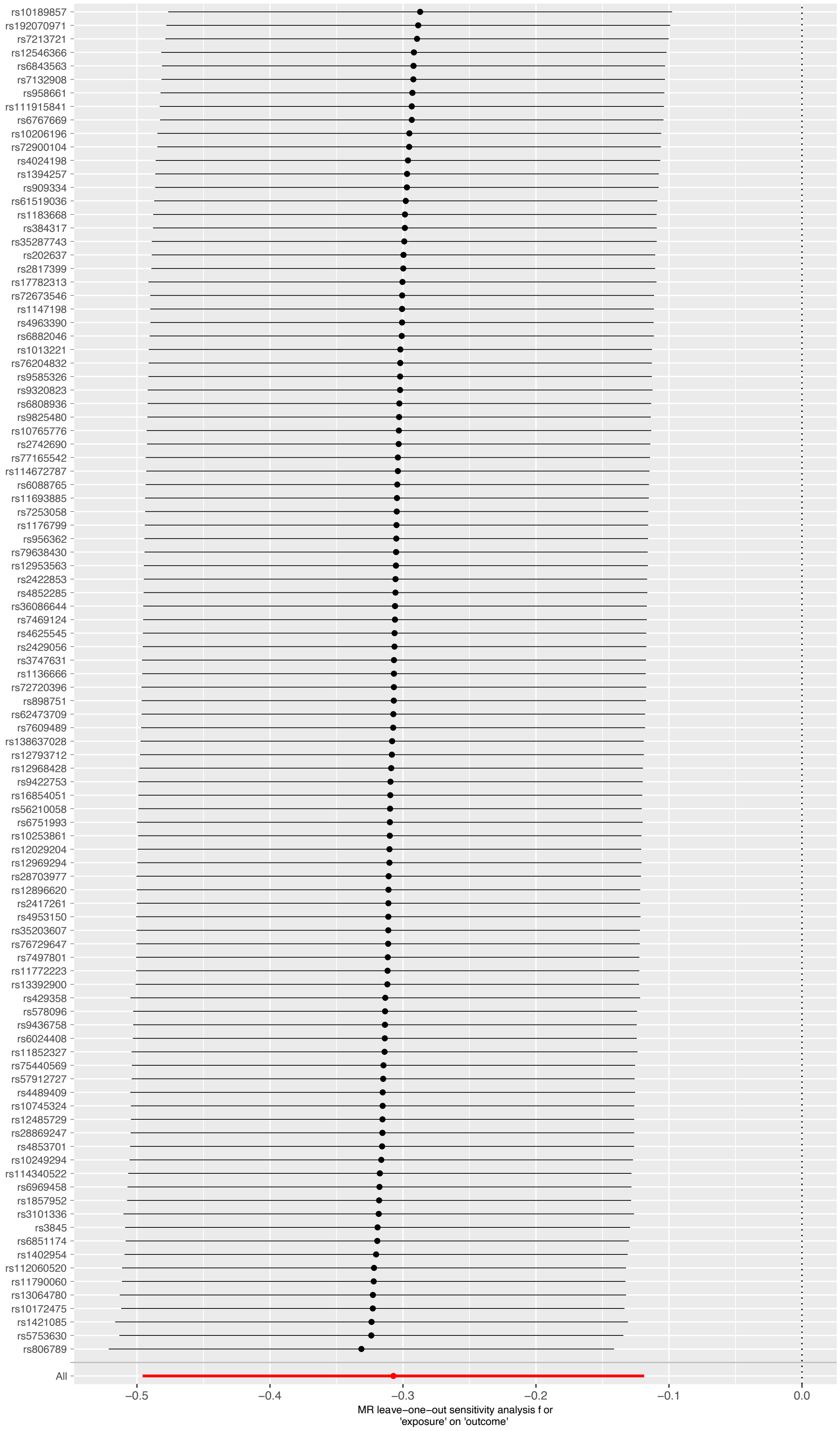

Supplementary Figure 1 Leave-one-out analysis of the effect of PC1 on BC risk

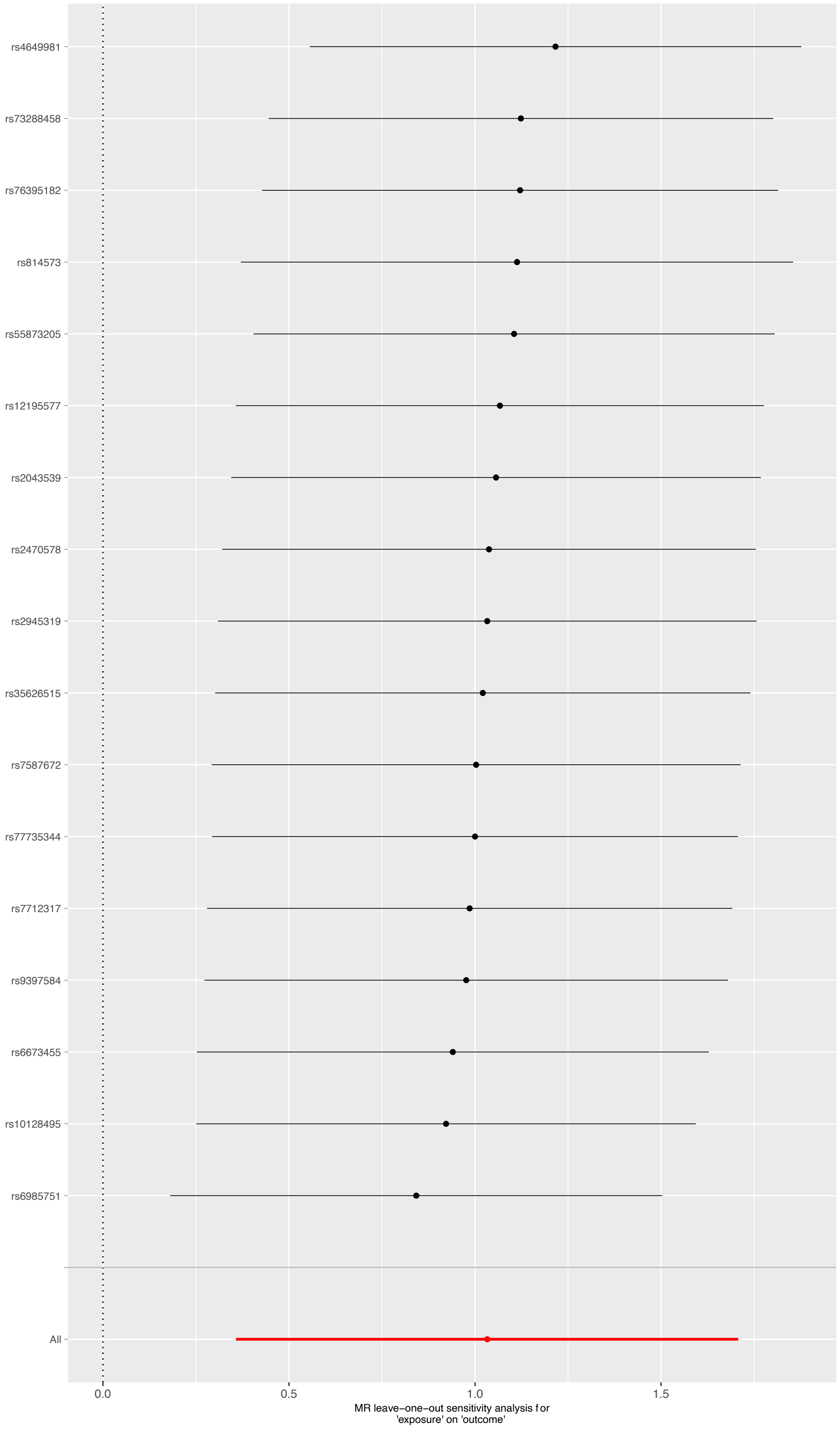

Supplementary Figure 2 Leave-one-out analysis of the effect of HPDP on BC risk
